# Automated detection of cylindrical structures in complex pipelines using iterative point cloud segmentation and high-precision fitting

**DOI:** 10.1038/s41598-025-30323-8

**Published:** 2025-11-27

**Authors:** Gengchen Cao

**Affiliations:** Tsinghua University - Anta Sports Fashion Joint Research Centre, Beijing, 100084 China

**Keywords:** Cylinder detection, Unorganized point clouds, Reverse engineering, Industrial pipelines, Computer science, Mechanical engineering

## Abstract

Cylinders are prevalent structural units in pipelines, and their accurate and robust detection from 3D scanned point clouds is crucial for rapid reverse engineering. Existing methods often impose restrictions on cylinder parameters, limiting their applicability in complex pipeline scenarios. To address this, we propose a novel method for automatically detecting cylinders from unstructured point clouds. Our approach involves iterative clustering segmentation to reduce data complexity, reliable candidate cylinder estimation using three-point random sampling, high-precision cylinder fitting, and multi-filtering mechanisms to minimize false detections. Experimental results on both simulated and real-world data demonstrate that our method achieves precision, recall, and F1 scores of 0.8727, 0.8090, and 0.8397, respectively, outperforming existing methods. This work showcases the potential of our approach for automating the reverse engineering design of complex pipelines. Project Web: https://github.com/GCCao/Cylinders_detection_Cao_V2.

**Supplementary Information:**

The online version contains supplementary material available at 10.1038/s41598-025-30323-8.

## Introduction

Pipeline systems play a crucial role in high-end equipment such as aerospace, shipbuilding, rail transportation, and energy engineering, serving as the essential components for fluid and gas transmission and system connectivity. Among these, complex pipelines with medium to large diameters are often manufactured using segmented assembly and welding techniques due to their high rigidity. However, during the manufacturing and assembly processes, unavoidable factors such as assembly errors, welding shrinkage, and material deformation can lead to discrepancies between the actual physical model and the original design model. These discrepancies can result in a decline in the performance of the pipeline system and may even pose safety risks. Therefore, high-precision scanning measurements and reverse modeling of complex pipelines after completion have become important methods for ensuring their quality and performance. Additionally, with the rise of digital twin technology, pipeline measurement and reverse modeling not only contribute to condition monitoring and fault diagnosis but also support predictive maintenance and system optimization, which are of significant importance for enhancing the reliability and operational efficiency of high-end equipment^1^.

**Fig. 1:**
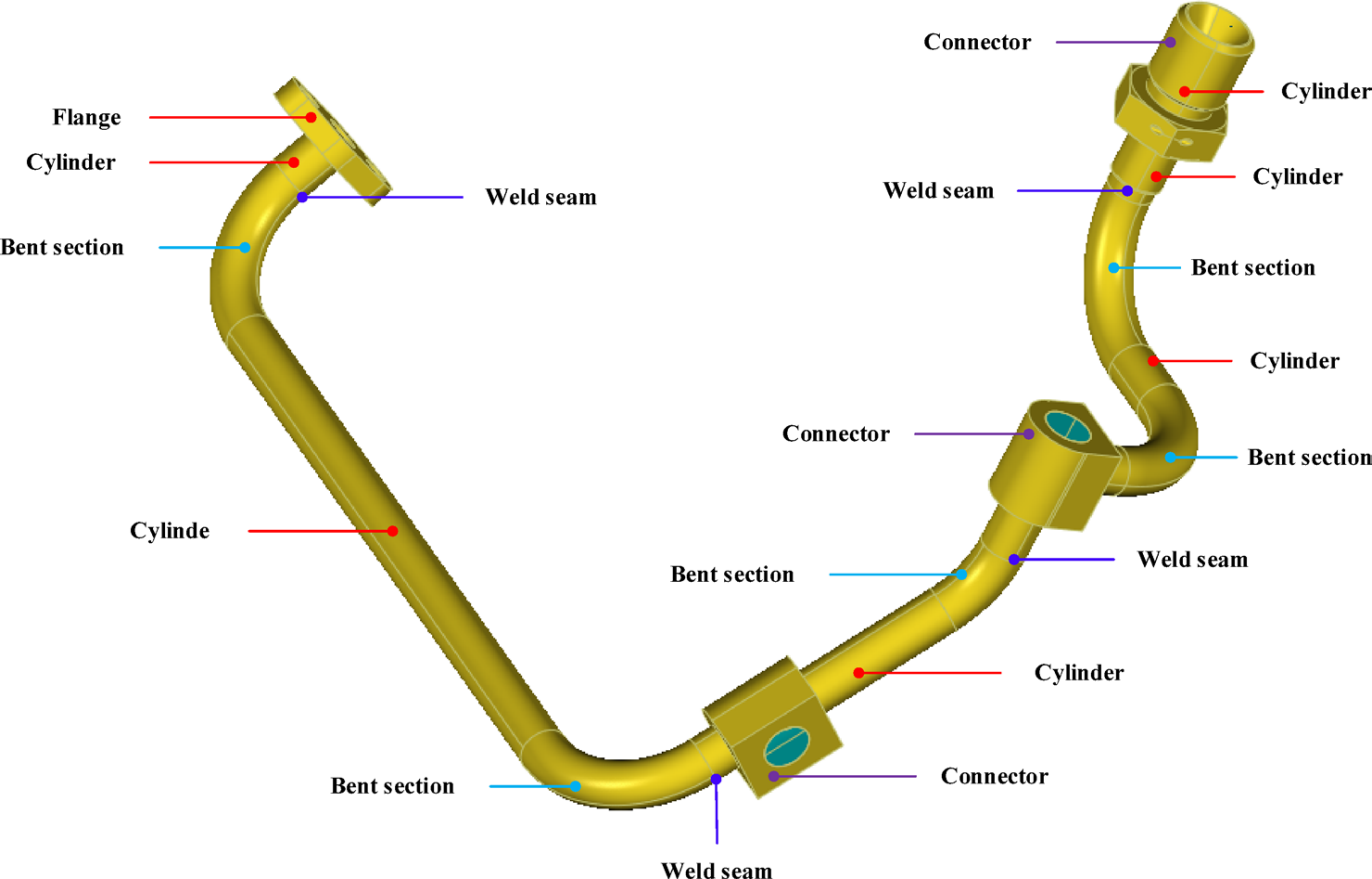
Example of complex aviation pipeline (The 3D image was generated by the authors using PolyWorks (Version MS2021, https://www.polyworks.com)).

In the field of high-end equipment manufacturing, complex pipelines typically consist of pipe bodies, bends, joints, and flanges, among which pipe bodies, joints, and flanges often contain cylindrical structures, as shown in Fig. 1. Currently, the three-dimensional digital modeling of completed pipelines in industry largely relies on human-computer interaction. Operators are required to manually identify and extract pipeline features using specialized software, followed by modeling. Even for experienced engineers, this process demands significant time and effort and is susceptible to subjective factors, making it difficult to meet the requirements for high efficiency and accuracy. With the rapid development of three-dimensional scanning technology, obtaining high-density and high-precision point cloud data has become more convenient, providing the possibility for automated reverse modeling of pipelines^2^. Cylinders are the most fundamental and common structural units in pipeline systems. The automatic and robust detection of cylinders from complex three-dimensional scanned point clouds is a critical step in achieving automated pipeline modeling. However, challenges are faced in the automatic detection of cylinders due to the unstructured nature of point cloud data and the complex layout of pipelines^3^. Some existing methods often impose restrictions on parameters such as cylinder orientation and aspect ratio, or they perform poorly in the presence of bends or flanges. Therefore, there is an urgent need for a detection method that is highly adaptable to pipeline point cloud data, possesses strong robustness, and does not impose special restrictions on cylinder parameters.

In this paper, a novel point cloud-based automatic detection method for cylinders is proposed, which aims to accurately detect cylindrical structures from unstructured scanned point clouds in complex industrial pipeline scenarios. The main contributions are as follows:

- An iterative clustering segmentation strategy for unstructured 3D scanned point clouds is proposed: This strategy enables the division of complex point cloud data into several sub-regions, reducing the complexity of data processing while increasing the likelihood that sampled points originate from the same cylinder. This provides a solid data foundation for subsequent cylinder estimation and overcomes the efficiency issues faced by traditional methods when processing large-scale point cloud data.
- A candidate cylinder estimation method based on three-point random sampling is introduced: In each subclass point cloud, a three-point random sampling algorithm is proposed for reliable estimation of candidate cylinder parameters. This method effectively enhances the reliability of initial estimates without imposing restrictions on parameters such as cylinder orientation and aspect ratio, making it suitable for the diverse cylinder detection in actual complex pipelines.
- A high-precision cylinder fitting algorithm is employed for accurate localization and validation: A high-precision cylinder fitting algorithm is utilized for the candidate regions, achieving a precise localization and parameter optimization of the cylinders, which significantly improves the accuracy of cylinder detection.
- A multi-filtering mechanism is designed to reduce the false positive rate of the algorithm: By introducing filtering mechanisms such as consistency detection and constraints on the angles between neighboring axes, the probability of false detections is further reduced, enhancing the robustness and reliability of the algorithm. Experimental results demonstrate that the proposed method achieves excellent performance in terms of precision, recall, and F1 score, proving its potential for application in the automated reverse engineering design of complex pipelines.

Section 3 details the main process of the pipeline cylinder detection method. Section 4 presents the experimental results of the proposed method. Finally, Sect. 5 discusses the conclusions and future work.

## Related work

Numerous cylinder detection methods have been proposed by researchers, which can be broadly categorized into the following types: methods based on RANSAC (Random Sample Consensus), methods based on Hough Transform, methods based on deep learning, and other methods. A detailed review of the relevant research work will be conducted based on the aforementioned classifications.

### Methods based on RANSAC

The fundamental concept of cylinder detection methods based on Random Sample Consensus (RANSAC) is to utilize a strategy that combines random sampling with model validation to reliably extract cylinder models from data characterized by significant noise and outliers. By iteratively refining the model, this method is capable of effectively ignoring outliers and noise, allowing for robust parameter estimation of the data and ultimately leading to the precise detection of the cylinder’s position and shape. This approach has demonstrated strong noise resistance and reliability when processing real-world point cloud data, and it is widely applied in cylinder detection tasks within fields such as computer vision and 3D reconstruction. In practical applications, the RANSAC algorithm is typically not used in isolation but is often combined with local feature information from the point cloud, such as normal vectors and curvature. These local features provide critical information regarding the shape and orientation of the point cloud surface, contributing to enhanced accuracy and robustness in model fitting.

In early research, Bolles et al.^4^ proposed a RANSAC-based model fitting method and applied it to detect cylinders from depth data. Subsequently, Schnabel et al.^5^ introduced a RANSAC-based shape detection algorithm capable of automatically identifying basic geometric shapes, including planes and cylinders, in 3D point clouds. The detection steps of the Schnabel method involve first determining the axis direction of the cylinder by computing the cross product of the normal vectors of two randomly sampled points. Then, the line projections of these two points onto a plane perpendicular to this axis are computed, with the intersection serving as the cylinder’s center; the radius is determined by the distance from the center to one of the points. Finally, candidate cylinders are validated using pre-set distance and normal vector deviation thresholds to ensure accuracy. However, the discriminative conditions used in the candidate cylinder estimation phase of this method are overly lenient, leading to a high false alarm rate by mistakenly recognizing the curved sections of pipes as cylinders. Moreover, the algorithm may overlook small-scale details or regions requiring fine segmentation, resulting in incomplete detection outcomes. Liu et al.^6^ proposed an adaptive reverse engineering approach that converts point clouds into CAD models. This method first employs the RANSAC algorithm to extract initial geometric primitives from the point cloud, including planes, spheres, and cylinders. To address the limitations of traditional RANSAC methods regarding parameter settings and sensitivity to noise, Liu introduced a strategy of histogram analysis of the orthogonal distances from points to the fitted primitives, facilitating adaptive parameter adjustment and error analysis. Jin et al.^7^ presented a rapid cylinder matching method in large-scale point cloud data. This method utilizes RANSAC and PCA (Principal Component Analysis) to estimate the central axis and radius of cylinders without prior steps such as noise filtering, normal estimation, or segmentation. By distinguishing between linear and curved regions and employing Catmull-Rom spline curve fitting, this approach significantly improves efficiency while maintaining the accuracy of traditional methods. However, the reliance on a known cylinder diameter when fitting ellipses may reduce matching accuracy in complex scenarios. Lee et al.^8^ utilized two user-selected points for initialization to approximate the corresponding pipe nozzle. Subsequently, the RANSAC algorithm is applied to fit the cylinder near the midpoint, and a neighborhood search is used to identify the set of points corresponding to the pipe. Finally, the point cloud is segmented based on these points to generate a pipe model. However, as the initial process of this method requires manual involvement, it sacrifices a degree of automation. Oh et al.^9^ proposed a method for detecting and reconstructing pipes from 3D point clouds. The method begins by extracting points on the cylindrical surface through the calculation of principal curvature and estimates the cylinder’s radius using histograms. Next, a RANSAC-based approach is employed to estimate the center and orientation of the cylinder while analyzing the connectivity between adjacent cylinders to determine how they connect in space to form a complete pipeline structure. This method does not require users to provide predefined parameters and can automatically identify and reconstruct pipes, demonstrating better performance than existing methods. However, the method is sensitive to sparse point clouds; when the cylinder’s radius is small or scanning is insufficient, the points in the point cloud may be inadequate for effective detection, leading to identification failure. Bergamasco et al.^10^ introduced a method for extracting cylinders without requiring point normals, treating the problem as a clustering task. Initially, two-dimensional ellipses are generated through random plane cutting, followed by RANSAC-based ellipse fitting to obtain candidate cylinders. The process then uses a game-theoretical inlier selection mechanism to extract the cylinders with the highest mutual support from the candidates. This method exhibits high robustness when dealing with noise and occlusions. However, the approach relies on assumptions about the cylindrical axes in the environment, which may impact accuracy in complex scenarios. The advantages of RANSAC-based cylinder detection methods include efficiency, the ability to handle high-noise data, and robustness against outliers. However, a notable drawback is the potential for false cylinder detection results, wherein the algorithm may identify cylinders that do not actually exist, a phenomenon referred to as the “false positive” issue in RANSAC. Furthermore, RANSAC is a non-deterministic algorithm, and its results may vary due to random sampling^11^.

### Methods based on Hough transform

The Hough transform enables cylinder detection in point clouds by mapping points that may belong to a cylinder into a parameter space. This space represents potential cylinder parameters like center, radius, and axis direction. Votes from points are accumulated in this space, and peaks indicate likely cylinder parameters. The transform leverages the geometric properties of cylinders, transforming a complex shape detection problem into a peak-finding problem in a higher-dimensional space.

Rabbani et al.^12^ proposed an efficient Hough transform method for the automatic detection of cylinders in point clouds. The approach begins with a 2D Hough transform utilizing a Gaussian sphere to estimate the cylinder’s axial direction, followed by a 3D Hough transform to estimate the position and radius of the cylinder. The density of cells on the Gaussian sphere impacts the accuracy of the cylinder’s directional estimation^13^. Additionally, the normal vector for each plane is used as a reference for estimating the cylinder’s axis. The estimation of the cylinder’s radius and center position is achieved through analysis of projected circles. Both plane and circle detection processes utilize the Hough transform. However, a drawback of this technique is that errors during the plane detection phase may propagate to the subsequent circle detection phase. Furthermore, to achieve optimal detection performance, researchers must adjust parameters for two different types of Hough transforms, which can be a cumbersome process. Patil et al.^14^ proposed improvements to the techniques described by Rabbani. By enhancing the Hough transform, they automatically detect the parameters of cylinders within point clouds and reconstruct 3D pipe models, thereby reducing computational space and time complexity while improving the accuracy of cylinder detection. However, the accuracy and robustness of detection are insufficient for cylinders with smaller radii in point cloud data. Díaz-Vilariño et al.^15^ introduced a method for the automatic detection and segmentation of columns from indoor architectural point cloud data. This method targets both circular and rectangular cross-sectional columns by rasterizing the point cloud data onto the XY plane and implementing a model-driven detection strategy based on Hough transforms. The study assumes that the floors and ceilings of buildings are parallel to the XY plane and that walls are aligned with either the X or Y axis. These assumptions ensure that the columns are vertical and their faces are parallel to the coordinate axes, which is necessary for correct rasterization and column detection steps but also limits the general applicability of the algorithm. Figueiredo et al.^16^ presented a robust and efficient framework for the rapid detection and recognition of cylindrical objects, suitable for consumer-grade RGB-D cameras. Innovations include a soft voting mechanism using curvature information to reduce the impact of outliers and noise, prioritizing the sampling of known directions, and employing deep learning classifiers to filter out non-cylindrical objects, in which case a Hough transform is used to obtain the cylinder parameters (axis, radius, and center). This method performs exceptionally well under conditions of noise and outliers, significantly improving recognition speed and accuracy by combining 2D and 3D data. However, the assumption that objects are located on planes may limit its applicability in practical situations. Hough transform-based cylinder detection algorithms exhibit several common drawbacks and limitations. First, these algorithms have high computational complexity, particularly when processing a large number of parameters in three-dimensional space, leading to significant increases in computation time and resource demands. Second, the performance of these algorithms is heavily dependent on parameter selection, such as the range of radii and thresholds, which typically need to be determined empirically or experimentally, thereby complicating their application. Although the Hough transform can handle partial occlusions to some extent, its detection performance significantly deteriorates in cases of severe local occlusion.

### Methods based on deep learning

Traditional methods often rely on predefined parameters and user intervention, which limits their application in complex scenarios. In recent years, deep learning-based techniques have emerged, offering more flexible and efficient solutions. These methods can automatically extract features from point clouds, identify cylinders, and demonstrate strong robustness when handling noise and irregular data. Cheng et al.^17^ proposed the DeepPipes architecture for pipeline detection. DeepPipes extracts features from point cloud data using convolutional neural networks and classifies them into different pipeline components. It combines density clustering and minimum spanning tree algorithms to generate a connected pipeline skeleton, ultimately optimizing the complete 3D pipeline model using the iterative closest point algorithm. This method is sensitive to noise in the initial classification and relies on subsequent processing steps to ensure consistency in component relationships and topology. Xie et al.^18^ introduced PipeNet, a deep learning network designed to automatically reconstruct building pipeline systems from point cloud data. The primary idea is to extract spherical patches from the original point cloud data, employing a local embedding module and dynamic scale manager to extract local features, perform semantic classification, and regress centerline nodes. Ultimately, the complete pipeline model is reconstructed through line fitting and graph-based connectivity analysis, converting the output into the BIM IFC 4.0 format. Although PipeNet does not require data preprocessing, simplifying the pipeline detection and reconstruction process, it faces challenges such as complex parameter tuning and sensitivity to scanning coverage angles and noise. Xu et al.^19^ improved the PointNet architecture by computing different types of features separately in the early stages and training them with independent networks. This work integrates traditional shape features (such as SHOT and rotational images) with deep learning networks, enhancing the feature representation of point cloud data. While deep learning-based cylinder detection methods show promise, they currently exhibit several limitations: memory constraints necessitate preprocessing downsampling, which may overlook small pipes; computational resources are limited when handling large-scale data; reliance on a large volume of labeled data, which is difficult to obtain in industrial settings; and limited generalization capabilities, as performance may decline when adapting to new data types.

### Other methods

Using planned 3D/4D models to constrain the search space for object extraction enhances the accuracy and efficiency of pipeline and flange extraction. The point cloud is registered with the planned model to leverage the known positions and orientations of flanges within the model, facilitating the rough extraction of potential pipeline and flange pairs from the point cloud data. Maalek et al.^20^ proposed a geometry-based method for recognizing cylinders and flanges in point cloud data. Their procedure includes registering the point cloud to the planned model coordinate system, coarsely extracting potential pipeline and flange pairs, extracting the main cylindrical surface from isolated points, filtering noise and labeling connected components, extracting flange planes, and estimating the centers and normal vectors of flanges. However, the limitations of this method include sensitivity to the size of the cylinder’s neighborhood and limited robustness under high noise and low point density conditions. Moreover, cylinder detection methods based on prior models heavily depend on accurate registration, requiring that the physical state of the object closely matches the design model; otherwise, achieving accurate registration becomes challenging. Araújo et al.^21^ proposed a connectivity-based cylinder detection method primarily designed for unorganized point cloud data. This method begins by projecting the point cloud onto a unit hemisphere from multiple directions (typically 100 directions) to detect circular projections, adjusting the projection directions using PCA. Subsequently, a neighborhood graph is computed to identify connected components, and a fast, robust circle recognition technique is applied to detect the circular projections. After identifying circles, outlier removal is performed, followed by a least squares fitting process to model the cylindrical surfaces. Finally, multiple connected components corresponding to the same cylinder are validated and merged. This method requires the ratio of the fitted cylinder’s height to its radius to exceed a certain threshold, which limits the detection of some flatter cylinders, such as those found on flanges. Moradi et al.^22^ estimated the normals from 2D depth maps and utilized the Maximally Stable Extremal Regions (MSER) as a feature detector for cylinder detection. The effectiveness of MSER is largely dependent on the selection of initial points. Furthermore, MSER primarily focuses on the stability of regions and their extremal properties, which may result in insufficient accuracy when detecting certain shapes, such as elongated cylinders. Recently, Du et al.^23^ proposed a cylinder extraction method combining multiscale prospecting and region growing. Through a five-stage process—point cloud downsampling, normal-vector preprocessing, multiscale core-region detection, iterative rough extraction, and length-constrained fine extraction—the method simplifies three-dimensional cylinder fitting into a two-dimensional circle fitting approach, thereby significantly improving efficiency. However, further optimization is still needed for handling complex topological structures. Markovic et al.^24^ proposed a method for simultaneously recognizing planes and cylinders, which primarily involves three stages for the identification and extraction of geometric elements: first, segmenting the point cloud to identify different planes and cylinders; second, merging overly segmented regions and estimating surface parameters; and finally, extracting the identified cylinders and planes. However, this method is mainly applicable to axis-aligned geometries and is inadequate for recognizing freeform surfaces. Wu et al.^25^ proposed QuadricsNet, a lightweight network that learns concise quadric representations for geometric primitives (e.g., cylinders, planes) in point clouds. Their method demonstrates efficient handling of sparse or noisy data through adaptive feature fusion, which is particularly relevant to industrial pipeline detection scenarios where cylindrical structures dominate. QuadricsNet demonstrates the potential of learning-based methods for primitive detection but does not specialize in industrial pipeline constraints (e.g., bend geometries). Surface defect detection is critical for ensuring pipeline integrity. Song et al.^26^ proposed MFANet, a multifeature aggregation network for few-shot defect segmentation on seamless steel tubes. Their work highlights the importance of cross-granularity feature fusion in industrial inspection tasks. While our focus is on geometric modeling rather than surface defect analysis, MFANet’s methodology underscores the potential for integrating structural detection (e.g., cylinder fitting) with surface quality assessment in future end-to-end inspection frameworks.

Based on the above analysis, the existing methods for cylinder detection are found to have the following limitations: First, most methods are sensitive to parameters and have limited generalizability. For example, RANSAC relies on random sampling, which can easily lead to false detections (such as misidentifying curved sections as cylinders). The Hough Transform requires predefined parameters such as radius ranges and orientation thresholds, making the process cumbersome. Meanwhile, region-growing methods, due to their fixed neighborhood sizes, struggle to adapt to the fitting of large-radius cylinders. Second, there are bottlenecks in computational efficiency. The high-dimensional parameter space of the Hough Transform can cause exponential growth in complexity. Third, data integrity is insufficient. Although deep learning methods can automatically extract features, they are limited by the scarcity of labeled data in industrial scenarios and the problem of missing small-diameter pipes. Finally, assumptions about the scene limit adaptability. Some methods depend on prior models (such as assuming that cylinders are vertically distributed) or strong geometric constraints (such as aspect ratio thresholds), making them incapable of handling inclined pipes or flat flange structures. Assumptions based on planar positioning also fail to cope with complex spatial layouts. These defects collectively restrict the application effectiveness of existing methods in complex industrial piping scenarios.

In response to the aforementioned issues, this study proposes a systematic solution: First, by employing iterative clustering segmentation and a tri-sampling strategy, the pre-defined constraints on cylinder orientation and size are eliminated, enabling adaptive detection of pipes with arbitrary spatial layouts. Second, a multi-level filtering mechanism is designed to effectively suppress false detections and interference from outliers. Third, a tri-sampling RANSAC strategy is adopted to reduce the number of invalid model fittings, balancing efficiency and accuracy in large-scale point cloud processing. Finally, the reliance on external data such as BIM/CAD model registration is minimized. Cylinder detection is achieved based solely on the geometric features of the point cloud, enhancing the universality of the method in scenarios where auxiliary information is lacking. This method realizes high-precision and robust cylinder detection in complex industrial piping systems, providing an efficient and reliable solution for automated reverse engineering.

## Method

**Fig. 2:**
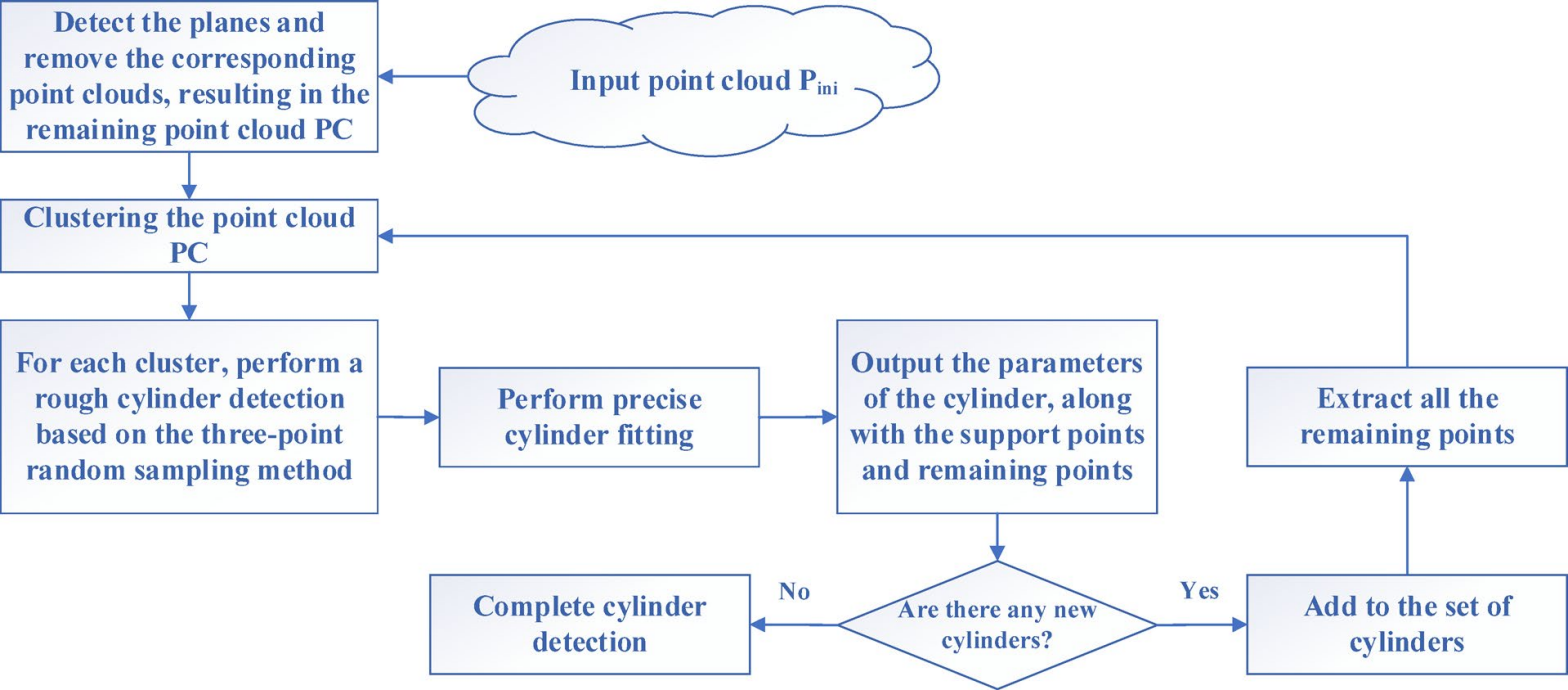
Our cylinder detection pipeline. The proposed cylinder detection method starts by clustering the initial point cloud into subsets. For each subset, candidate cylinders are detected using a three-point random sampling technique, yielding initial parameter values. After precise fitting of cylinders, the supporting points are calculated, and the cylinders are removed. The remaining points are then stored for further clustering, repeating the process until no new cylinders are found.

Cylinders can typically be represented using seven parameters:*a*, *b*, *c*, *d*, *e*, *f*, *r*, where **c**(*a*,*b*,*c*) denotes a point on the axis of the cylinder,**V**(*d*, *e*, *f*) represents the direction vector of the cylinder’s axis, and *r* is the radius of the cylinder. The proposed cylinder detection methodology is illustrated in Fig. 2. The process begins with plane detection on the initial point cloud P_ini_ (see^5^, removing the planar point cloud to reduce interference points. The remaining point cloud PC is then clustered into *Nc* subsets of point clouds. For each clustered subset, a candidate cylinder detection is performed using a three-point random sampling method (described in Sect. 3.2), which provides initial values for the cylinder parameters. Following this, precise cylinder fitting is carried out (described in Sect. 3.3) to obtain the supporting point cloud C_k_. The support points for C_k_ are then calculated, after which C_k_ is removed, and the remaining points are retained. Once all clustered subsets have been processed, all remaining points are stored as a new point cloud, which is then subjected to further clustering. This iterative process continues until no new cylinders are found. The details of this process can be elaborated in Algorithm A1.

**Figure:**
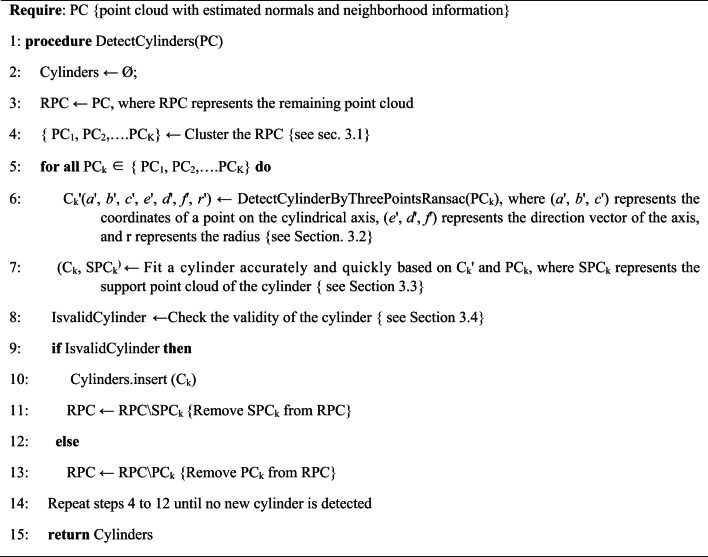
**Algorithm 1** CylindersDetection.

### Clustering the point cloud

To provide clarity, the supporting point cloud of the cylindrical shape is first defined. In this study, the supporting point cloud of a cylinder is characterized as the set of all points that meet two conditions: (1) the points closely surround and geometrically form the surface of the cylinder, and (2) the normal vector at each point is approximately perpendicular to the cylinder’s axis. The scanned point cloud of pipelines often contains multiple cylindrical shapes. As the cylindrical detection process progresses, the supporting points of the detected cylinders are gradually removed, resulting in the remaining point cloud forming multiple discrete regions. Each region contains different cylinders, which necessitates that sampling points should not originate from different discrete regions. However, during the random sampling process, points are generated randomly, which may lead to samples being sourced from disparate point cloud regions. To address this issue, clustering of the remaining point cloud is proposed as a reasonable solution, as it can enhance the probability that the sampling points are derived from the same cylinder, thereby reducing the number of iterations and improving detection efficiency. This method is crucial for the detection of cylindrical shapes within large-scale point clouds.

In this context, a KD-tree-based point cloud clustering method is introduced to facilitate the rapid and accurate grouping of point cloud data. The KD-tree serves as an efficient spatial indexing data structure, suitable for searching and partitioning high-dimensional spaces^27^. When compared to traditional octrees, the KD-tree offers superior performance and flexibility. Initially, a KD-tree is utilized to perform spatial indexing on the preprocessed point cloud. During the construction process, the space is recursively divided into subspaces until predetermined termination conditions are satisfied. A distance measure is defined to calculate the distance between points, with Euclidean distance employed as the metric in this study. Clustering is then conducted in a bottom-up manner, beginning from the leaf nodes of the KD-tree. For each node, the maximum distance between points in its child nodes is calculated. If this distance is found to be less than the threshold T_cluster_, the corresponding points are merged into a single cluster; otherwise, clustering proceeds for the child nodes. The preliminary clustering results are subsequently optimized by removing outliers and merging clusters that are excessively close together to enhance clustering accuracy and robustness. Through point cloud clustering, the search range for cylinders can be narrowed, thereby creating improved conditions for candidate cylinder detection.

The proper selection of the threshold T_cluster_ is of paramount importance, as it directly affects both the completeness of the clustering result and the accuracy of the segmentation. An excessively low threshold may cause a single object to be divided into several sub‑clusters, whereas an excessively high threshold may merge distinct objects erroneously, thereby impairing subsequent processing. The threshold must therefore be large enough to encompass the distance variations within the same cluster while still excluding points that belong to different clusters. Close observation reveals a strong correlation between the inter‑point distances and the threshold value. To this end, the following procedure is proposed for determining the distance threshold T_cluster_. First, n points are randomly selected from the point cloud PC. For each sampled point, the distances to its k nearest neighbours are computed, yielding n×k distance values. Second, all distances are sorted in ascending order and the median d_M_ is extracted. Finally, the threshold is defined as an appropriate multiple of this median, i.e. T_cluster_ = η·d_M_. By relying on the median of the neighbourhood distances, the local density of the point cloud is captured statistically, enabling an adaptive determination of the segmentation threshold that balances computational efficiency with clustering accuracy. The scaling factor η is typically chosen within the range [1.5, 3.0]. In a Poisson point process, a threshold of T_cluster_≥1.5d_M_ covers most intra‑region neighbour distances, whereas the larger inter‑region gaps generally exceed 2–3 d_M_. To accommodate irregularities such as local sparsity, sensor drop‑outs, and minor registration errors, while still minimising the erroneous merging of genuinely separate regions, a uniform value of η = 3 is adopted in all the experiments presented in this study. A moderately larger neighbourhood size, k∈^20,30^, reduces the variance while still maintaining locality. Choosing the number of random samples n within the interval [300, 1000] typically limits the estimation error to below 10%. In this study, k is fixed at 20 and n at 300.

In this paper, an iterative clustering strategy is employed to address the challenge of multiple interwoven and occluded cylinders in complex pipeline point clouds. Unlike traditional static clustering methods, a dynamic updating mechanism is adopted here: after each cylinder detection, the supporting point cloud is actively removed, and the remaining point cloud is re-clustered using the KD-tree algorithm to generate new sub-regions. This closed-loop process of “detection-removal-reclustering” significantly reduces data complexity, ensuring that each processing object is a locally distinct point cloud with clear geometric features, thus avoiding the ineffective sampling caused by globally mixed data in traditional methods.

### Candidate cylinder detection based on Three-Point random sampling

It is well established that a more accurate initial estimate of cylindrical shapes increases the probability of locating valid cylinders, thereby enhancing detection efficiency by reducing unnecessary cylinder fitting operations that are often time-consuming. In Schnabel’s cylinder estimation method^5^, two points along with their corresponding normal vectors are employed. This method is susceptible to producing erroneous results and is relatively inefficient. The primary reason for this limitation is the insufficient auxiliary judgment information provided by the two points and their normal vectors. To address this issue, a candidate cylinder estimation technique involving three points and their normal vectors is proposed in this study. The advantage of this approach lies in the ability to generate multiple intersection points and radii through various combinations, enabling the early elimination of unreasonable cylindrical shapes based on their consistency. This process reduces the occurrence of false positives while avoiding excessive large-scale distance calculations in subsequent steps, thereby improving detection efficiency. Specifically, from a geometric perspective, lines defined by the normal vectors at any point on the cylinder’s surface intersect with the cylinder’s axis. When three surface points are selected, three corresponding intersection points can be derived based on a pairwise intersection strategy. If these intersection points are projected onto a plane aligned with the cylinder’s axis, their projected points will approximate coincidence. This observation can be utilized to assist in determining whether the three randomly sampled points are situated on the surface of the cylinder.

**Fig. 3:**
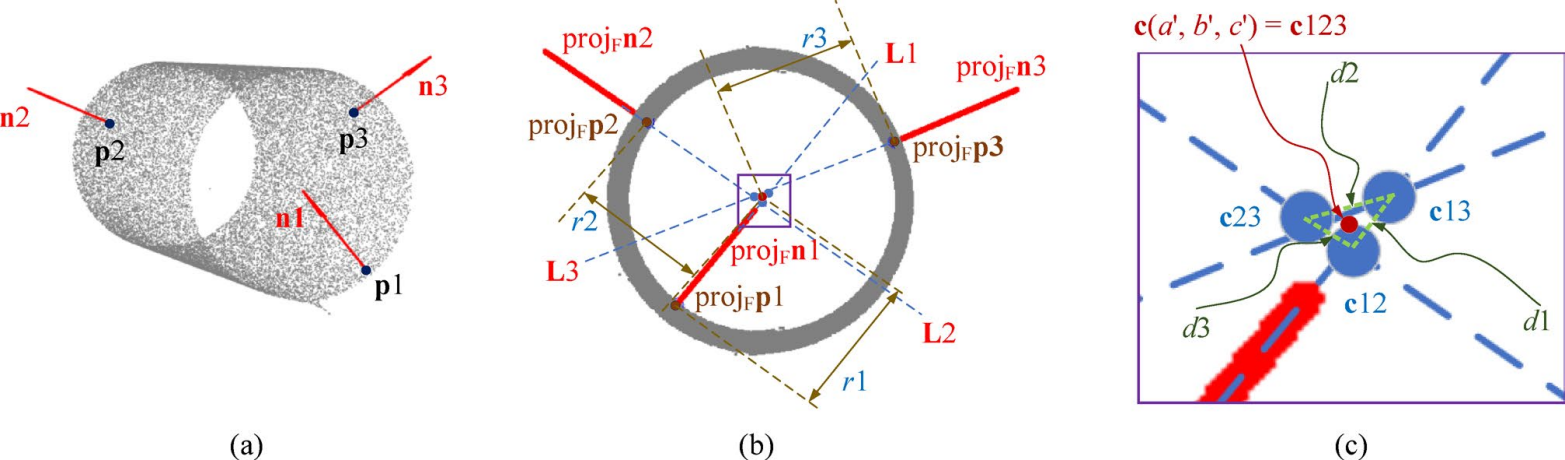
Visualization breakdown of the calculation process of Algorithm 2. (a) the three randomly selected points and their normal vectors. (b) the projections of the three points and their normal vectors on plane F. (c) an enlarged view of the intersection points obtained from the pairwise intersections of the three lines after projection。.

**Figure:**
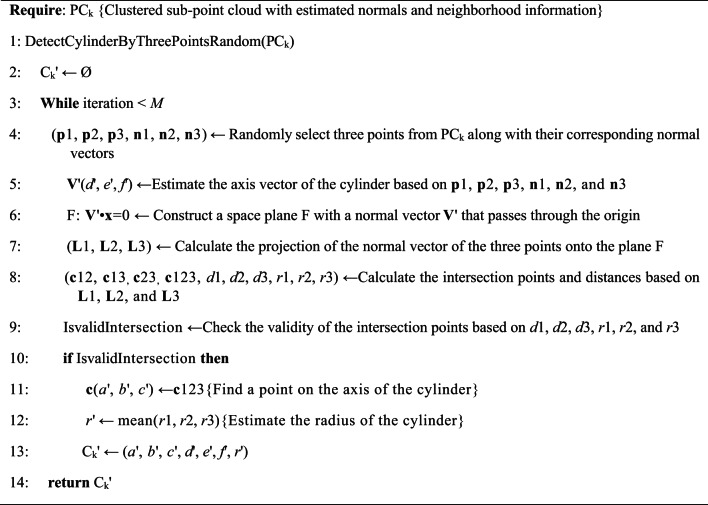
**Algorithm 2** Candidate cylinder detection based on the three-point random sampling method.

The proposed candidate cylinder detection method based on three-point sampling is outlined in Algorithm 2. It works as following: First, in the point cloud PC_k_, three points are randomly sampled, designated as **p**1, **p**2, and **p**3, and their normal vectors **n**1, **n**2, and **n**3 are obtained, as shown in Fig. 3(a). Subsequently, the axis vector of the cylinder is estimated. The cross product of the normal vectors of any two points is calculated to yield three cross product vectors **V**’12, **V**’13, and **V**’23. If the angle between any two cross product vectors is less than the threshold *T*_*cross*_ (*T*_*cross*_ is set to 5 degrees) it is inferred that the selected three points may lie on the cylinder, and the initial axis vector of the cylinder is calculated as, **V**‘(d’,e’,f’) = mean(**V**’12, **V**’13, **V**’23). A spatial plane F, which is perpendicular to the cylinder axis, is constructed such that, **V**’•**x** = 0. Points **p**1, **p**2, **p**3, and their normal vectors **n**1, **n**2, and **n**3 are projected onto F, resulting in proj_F_**p**1, proj_F_**p**2, proj_F_**p**3, proj_F_**n**1, proj_F_**n**2, and proj_F_**n**3, as shown in Fig. 3(b). Using proj_F_**p**1 and proj_F_**n**1, a parametric line **L**1: proj_F_**p**1 + t proj_F_**n**1 can be determined. Similarly, lines **L**2 and **L**3 can be obtained. Because **L**1, **L**2, and **L**3 are confined within the same plane F, intersection operations are conducted among them. The intersection points of any two lines among **L**1, **L**2, and **L**3 are computed to obtain points **c**12, **c**13, and **c**23. The distances between any two of those intersection points are then calculated to obtain *d*1, *d*2, and *d*3. Let **c**123 represent the centroid of **c**12, **c**13, and **c**23, as shown in Fig. 3(c). Next, the distance between **c**123 and proj_F_**p**1 is computed to derive *r*1, while *r*2 and *r*3 are obtained similarly. If the three randomly sampled points are located on the same cylindrical surface, *r*1 is expected to be approximately equal to the physical radius of the cylinder, and **c**12, **c**13, and **c**23 should converge to approximately the same point. Thus, the aforementioned clue is utilized to assess the validity of the intersection points and the radius. If the difference between any two of *d*1, *d*2, and *d*3 is found to be less than the threshold *T*_*dis*_, and the difference between any two of *r*1, *r*2, and *r*3 is also less than the threshold *T*_*dis*_, the intersection points are deemed reliable, suggesting a high likelihood that the three sampling points lie on the same cylinder surface. Finally, C_k_’ is outputted as a valid candidate cylinder. Should no valid cylinder be identified, three points are randomly selected again, and the verification process is continued until the maximum number of iterations is reached.

As the radius of the cylinder is increased, the convergence of the intersection points is observed to worsen. Consequently, it can be stated that the value of *T*_*dis*_ is associated with the radius of the cylinder; as the radius is increased, *T*_*dis*_ is also found to increase. Given the fact that *T*_*dis*_ is known to increase slowly with an increment in radius, a logarithmic polynomial is employed in this paper to represent the relationship between *T*_*dis*_ and the radius *r*:

1 $$\:{T}_{dis}={\lambda\:}_{1}{(\mathrm{l}\mathrm{o}\mathrm{g}(r\left)\right)}^{3}+{\lambda\:}_{2}{\left(\mathrm{log}\left(r\right)\right)}^{2}+{\lambda\:}_{3}\mathrm{log}\left(r\right)+{\lambda\:}_{4}\:\:\:\:\:\:\:\:\:\:\:$$

**Fig. 4:**
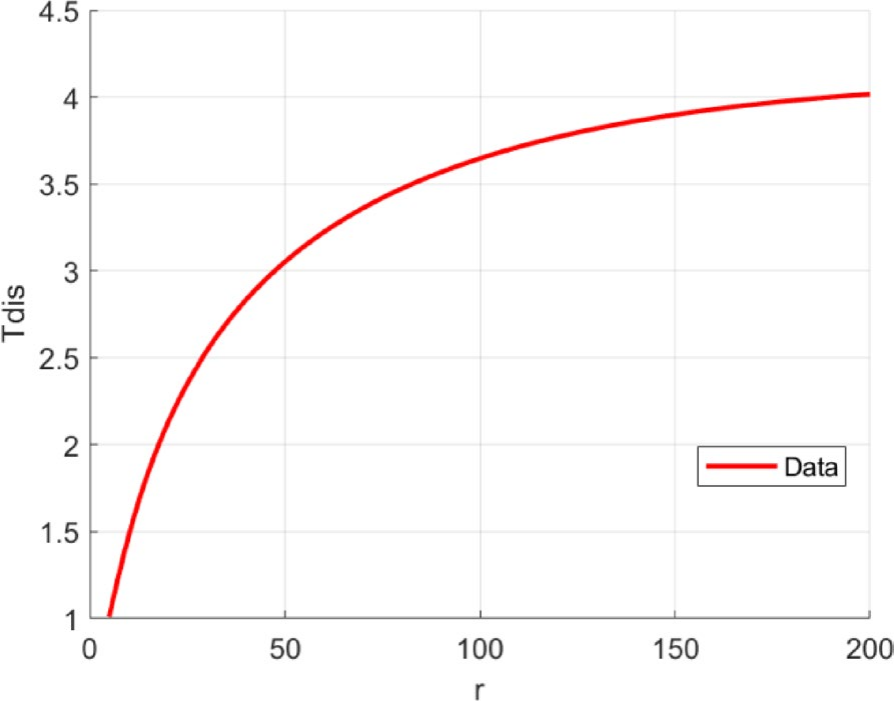
The curve of the distance threshold *T*_*dis*_ as a function of the radius *r*.

Here, the values of $\:{\lambda\:}_{1}$, $\:{\lambda\:}_{2}$, $\:{\lambda\:}_{3}$, and $\:{\lambda\:}_{4}$ are − 0.0612, 0.6109, −1.0069, and 1.3034, respectively, and they are obtained from experiments. The curve of Tdis with respect to r is shown in Fig. 4.

The setting of the maximum iteration count, *M* in Algorithm 2, is not considered arbitrary. If it is set too small, the identification of three points that meet the requirements cannot be guaranteed; conversely, if it is set too large, the iteration time will be increased. In fact, the value of *M* is related to the total number of points in the point cloud PC, the number of cylinders, and the number of points corresponding to each cylinder. To achieve a more accurate estimation of *M*, a detailed derivation of *M* is provided in this paper, as referenced in Appendix A.

### Fit a cylinder accurately and quickly

Once a candidate shape C_k_’ for a cylinder is identified, accurate cylinder fitting must be conducted for the ultimate acceptance of the cylinder. The least squares-based cylinder fitting method has been widely utilized^28^, with a focus on optimizing the geometric error of the candidate shape. Recently, modifications to the iterative process of this method have been implemented by Lin^29^., and editable MATLAB code has been made available. The mathematical formulas and workflow of the modified method are detailed on the corresponding webpage. In order to enhance the reader’s understanding, the analytical expression of the cylinder is derived in this paper (see Appendix B). In Lin’s method, if the point cloud is found to contain outliers, the program proceeds to the next iteration, excluding these outliers from subsequent calculations until none remain. Outliers are detected by calculating their residuals, which is greater than 3 times the sigma. Given the superior performance of Lin’s method in terms of accuracy and efficiency, this method is adopted for cylinder fitting in the current study.

When the point cloud data is very large, the fitting of cylinders is still regarded as a time-consuming process. It has been observed that, in the point cloud data of cylinders, once the number of points exceeds a certain threshold, the improvement in fitting accuracy becomes negligible. Consequently, data simplification techniques, such as random downsampling, can be employed to select an effective subset of points that retains essential geometric information while reducing data volume and improving the running efficiency of fitting algorithms. Based on the aforementioned observations, modifications to the input conditions of the Lin’s algorithm have been proposed in this paper to further accelerate the fitting process. It is stipulated that if the number of points corresponding to each cylinder exceeds *T*_*fit*_, the point cloud will be randomly downsampled to *T*_*fit*_ points. The value of *T*_*fit*_ has been derived from experimental validation and statistical data. For industrial pipelines, which typically contain multiple cylinders, usually ranging from 3 to 40, the upper limit of the number of cylinders is taken as the basis for calculation, meaning that the entire input point cloud is downsampled to 40*T*_*fit*_ points before cylinder fitting, while point cloud with fewer than 40*T*_*fit*_ points is left unchanged. In this paper, *T*_*fit*_ is set to 5000.

After obtaining the parameter model C_k_ of the cylinder using the fitting method, the distance *d* from any point **p** in PC_k_ to the surface of the cylinder C_k_ is calculated (see Appendix B), along with the angle *θ* between the normal at that point and the axis of the cylinder. If *d* < 3sigma and abs(*θ* − 90) < 10 degrees, then the point is considered a supporting point of the cylinder. All supporting points form the supporting point set SPC_k_ for the cylinder.

### Check the validity of the cylinder

**Fig. 5:**
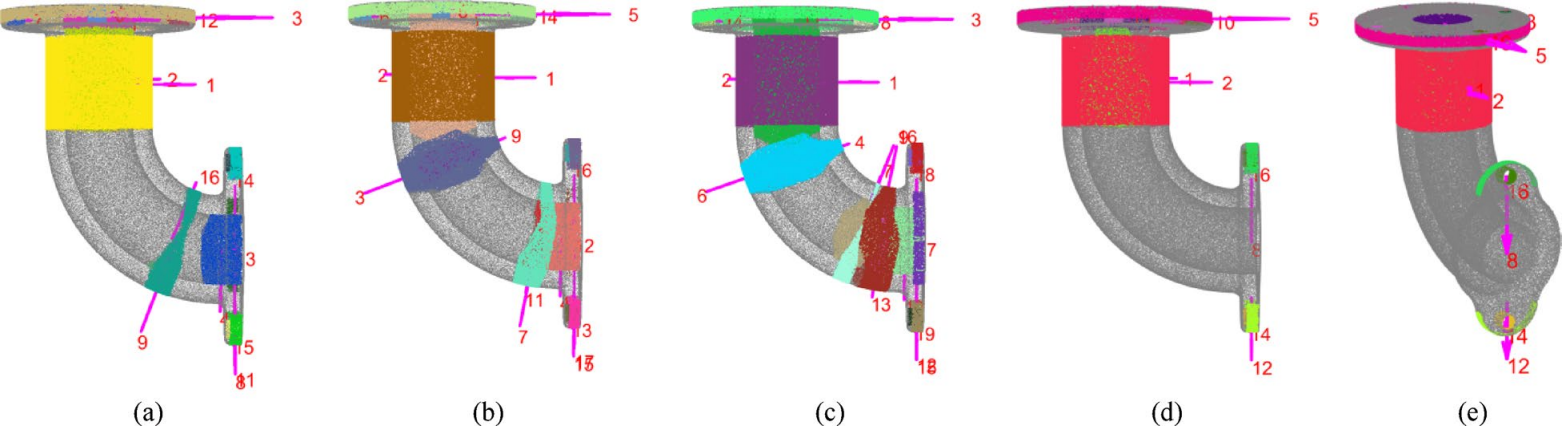
Verifying cylinders using multiple detection consistency and nearest neighbor axis angle constraints. The occurrence of false cylinders in multiple independent detections exhibits a certain level of randomness, indicating that they often do not consistently appear in the same position. This observation provides a basis for using consistency constraints in multiple detections. (a)-(c) Visualization of the results from three independent detections. (d)-(e) The final valid cylinders from two perspectives.

The aforementioned method cannot guarantee that all detected cylinders are valid. For instance, from a differential perspective, a bent section of a pipeline may be viewed as being composed of multiple short cylinders. Theoretically, the detection of multiple false cylinders at the bend is possible. The height-to-diameter ratio of false cylinders that appear in the curved sections is usually smaller than that of true cylinders, however, exceptions exist. For example, the side of a pipeline flange is composed of cylindrical shapes and exhibits a small height-to-diameter ratio. Therefore, false cylinders cannot be simply eliminated based on constraints related to the height-to-diameter ratio. Nevertheless, some favorable clues warrant consideration. Numerous experiments have revealed that the occurrence of false cylinders in two independent detections exhibits a certain level of randomness, indicating that they often do not consistently appear in the same position, as illustrated in Fig. 5. Additionally, aside from having a smaller height-to-diameter ratio, the angle between the axis of false cylinders and the axis of the nearest neighboring cylinder is typically neither parallel nor perpendicular, which significantly differs from the cylinders associated with the side of the pipeline flange.

Based on the observations described above, a method for checking the validity of cylinders is proposed in this paper, with its pseudocode shown in Algorithm 3, which is aimed at reducing false cylinders through the application of multi-detection consistency and nearest-neighbor axis angle constraints. The fundamental principle is as follows: three independent cylinder detections are performed on the input point cloud PC, resulting in three cylinder sets Cylinders1, Cylinders2, and Cylinders3. For any cylinder Ci within Cylinders1, if cylinders Cj and Ch can be identified in Cylinders2 and Cylinders3, respectively, such that the axes of Ci, Cj, and Ch are closely aligned, and the distances from the center of Ci to the axes of Cj and Ch are both below the threshold *T*_*dis*_, then cylinder Ci may be considered a candidate valid cylinder; otherwise, it is classified as a false cylinder. The set of cylinders obtained following the multi-detection consistency check is denoted as Cylinders_multi_. For any cylinder Ck in Cylinders_multi_, it can be assumed that the nearest cylinder to the center of Ck within Cylinders_multi_ is Ck_neigh_. If the angle *β* between the axes of Ck and Ck_neigh_ meets the criteria *β* < *T*_*β*_ or abs(*β* − 90) < *T*_*β*_, then Ck can be regarded as a final valid cylinder. All valid cylinders are aggregated to form the set Cylinders. It is noteworthy that the number of detections in the multi-detection consistency determination is allowed to exceed three, an increased number of detections means that fewer false cylinders are accepted, but it will result in increased computation time. For all results shown in the paper, the angular tolerance *T*_*β*_ is set to 2 degrees.

**Figure:**
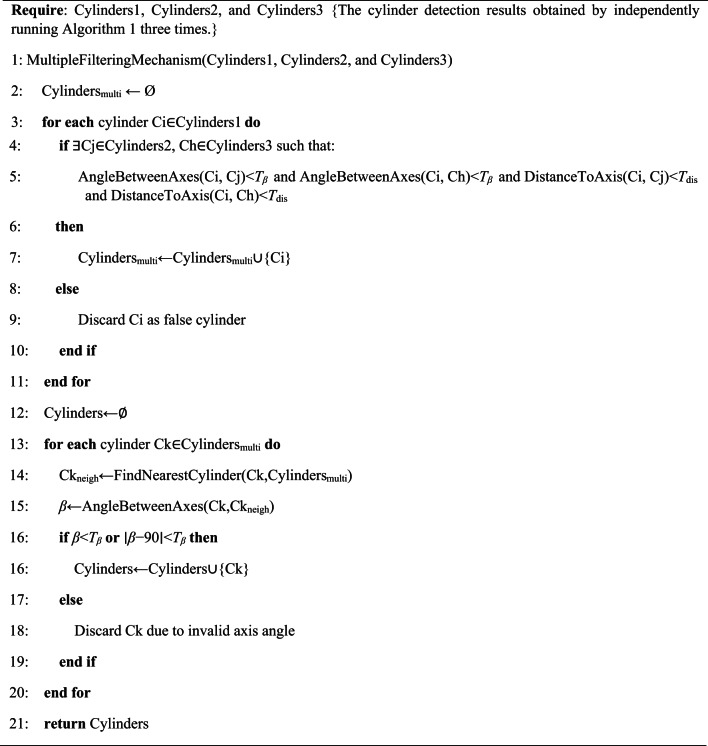
**Algorithm 3** Verifying cylinders using multiple detection consistency and nearest neighbor axis angle constraints

## Results

In order to verify the effectiveness and superiority of the proposed method, it will be evaluated through experiments. This section will detail the experimental platform, the selection of datasets, the definition of evaluation metrics, and the choice of comparative methods, in order to comprehensively analyze the performance of this method in both simulated data and practical applications. The experiments were conducted on a standard DELL desktop computer with the following configuration: 11th Gen Intel(R) Core(TM) i5-11400 F processor with a frequency of 2.60 GHz, 16 GB DDR4 RAM, and an NVIDIA GeForce GT 730 graphics card. The operating system used was Windows 10 Professional.

### Experimental data

**Fig. 6:**
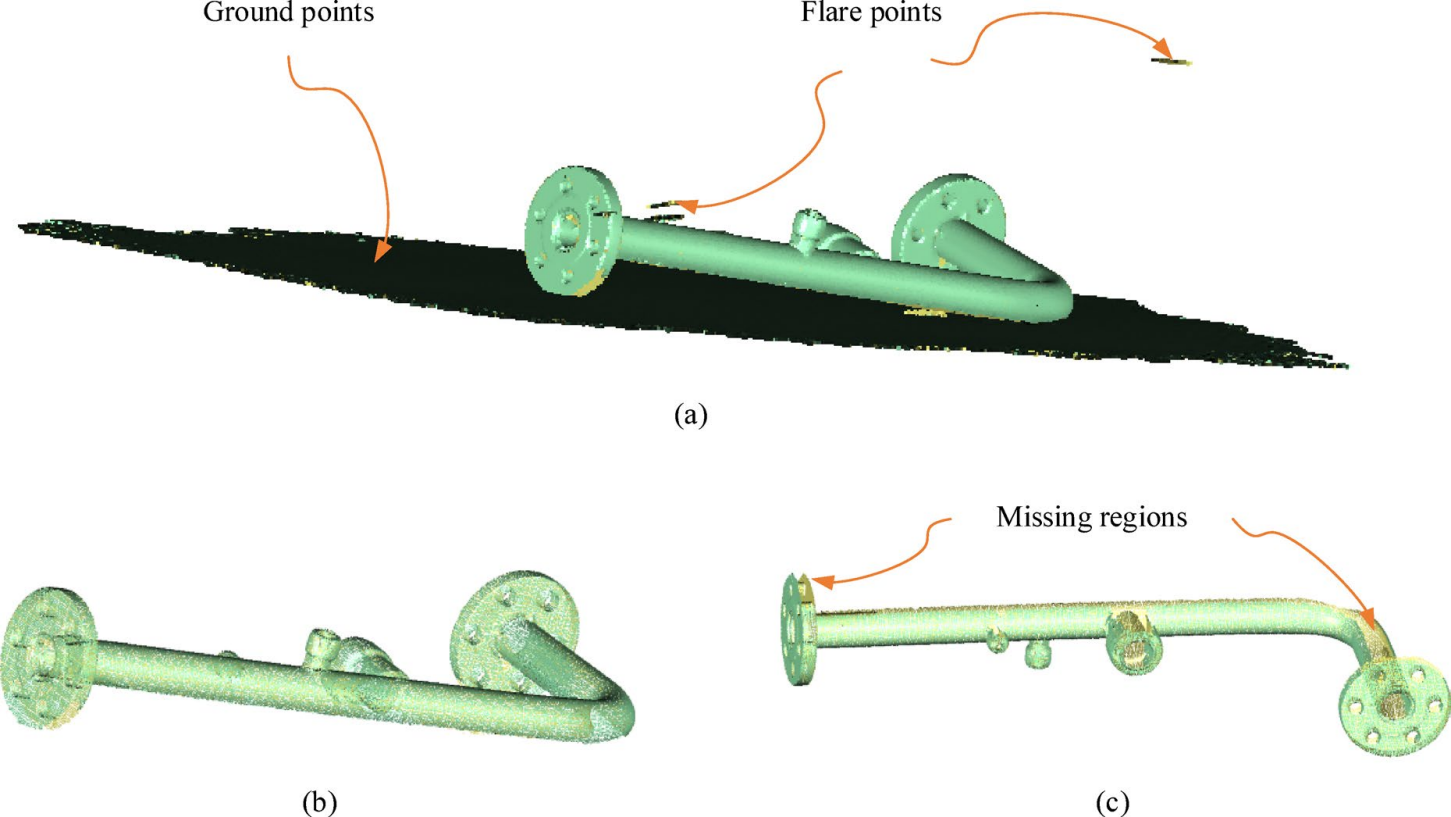
Interfering point clouds in pipeline scanning and the effects of preprocessing. (**a**) The scanned point cloud contains ground points and flare points. (**b**) The pipeline after removal of interfering points. (**c**) Missing regions in the point cloud caused by occlusion. (These 3D images were generated by the authors using PolyWorks (Version MS2021, https://www.polyworks.com)).

For the experiment, two types of datasets were carefully selected: one derived from simulated data and the other from actual collected data. Simulated Dataset: In order to verify the algorithm’s performance under ideal conditions, multiple point cloud data of pipelines were generated through computer simulation. These simulated data included cylinders with known dimensions, positions, and orientations, as well as interference objects of other geometric shapes. Measured Dataset: To validate the effectiveness of the algorithm in practical applications, point cloud data were collected from real industrial pipelines using the Hexagon HyperScan Super 3D scanner and the Gom Atos Q scanner. Both the HyperScan Super and Atos Q scanners possess high precision and high-speed acquisition capabilities, enabling the acquisition of high-quality point cloud data for complex-shaped pipelines. In the experiments described in this study, the pipeline is placed on the ground surface prior to scanning to ensure stable acquisition of scan data. Consequently, ground point clouds are inevitably included in the scan results. Additionally, due to interference from ambient stray light, flare-induced point clouds may appear in the camera images, as illustrated in Fig. 6(a). Although the proposed method demonstrates robust performance against interference point clouds, their significant proportion consumes substantial computational resources and severely degrades detection efficiency. Fortunately, the ground surface can typically be approximated as a planar structure, and flare-induced point clouds are generally isolated. These characteristics enable their removal through straightforward software operations. To address this, the Polyworks software’s “Selection Unit” function is utilized for interference point cloud removal. Specifically, the volume selection mode is activated, and the brush tool is employed to manually select interference regions. After deletion of these artifacts, a purified point cloud of the pipeline surface is obtained. The post-processing results are presented in Fig. 6(b).

Additionally, for pipelines placed on the ground, the side close to the ground is occluded, resulting in missing scan data (see Fig. 6(c)), which can affect the recall and precision of cylinder detection. To obtain complete scan data, the occluded side needs to be scanned additionally. The method adopted in this study involves flipping the pipeline after scanning one side and rescanning the accessible areas for the sensor. Subsequently, the point cloud data from the two scans are registered to construct a complete pipeline dataset. To ensure registration accuracy, each scan should cover as much overlapping area as possible. In this study, Polyworks software was used to register the scan data from both sides. The specific steps are as follows: First, the two datasets were aligned to an appropriate view, and at least three pairs of corresponding points in the overlapping area were manually anchored for coarse registration using point-to-point matching. Next, an iterative best-fit process was performed on the data points until the matching error converged, completing the fine registration. Finally, the two registered datasets were merged to obtain the complete pipeline point cloud.

The measured data contained pipelines of various sizes and materials, including aluminum alloy, stainless steel, and titanium alloy. The scanning scenes were set in a workshop environment, where various uncontrollable factors, such as lighting variations, surface reflections, and environmental noise, made the evaluation of the algorithm more representative of actual applications. Table 1 presents the number of sampled points, the number of cylinders, and the scanning sensors used for all pipelines, where Pipeline 1 to Pipeline 4 represent simulated pipelines and Pipeline 5 to Pipeline 9 represent actual pipelines.

**Table 1:** Dataset information: dataset name, number of cylinders, and source (sensor).

	Dataset name	# Points	# Cylinders	Source (sensor)
**Simulated dataset**	Pipleline1	560,034	12	
	Pipleline2	1,236,428	22	
	Pipleline3	940,233	15	
	Pipleline4	1,080,885	21	
**Real dataset**	Pipleline5	2,120,487	35	HyperScan Super
	Pipleline6	1,211,526	22	Atos Q
	Pipleline7	2,099,111	29	Atos Q
	Pipleline8	766,674	14	HyperScan Super
	Pipleline9	1,241,950	8	HyperScan Super

### Evaluation metrics

To objectively assess the detection performance of the algorithm, three commonly used evaluation metrics were employed: *Precision*, *Recall*, and *F1-Score*, to quantify the analysis of the detection results.

#### Precision

*Precision* is defined as the ratio of the number of correctly detected positive samples to the total number of positive samples detected by the algorithm.

2 $$\:Precision=\frac{true\:positive}{true\:positive+false\:positive}\:\:\:\:\:\:\:\:\:\:\:\:\:\:\:\:\:\:\:\:\:\:\:\:\:\:\:\:\:\:$$

where *true positive* refers to the correctly identified positive samples, and *false positive* refers to the incorrectly identified positive samples. *Precision* reflects the accuracy of the algorithm’s detection results, indicating how many of the detected objects are actual targets. A higher *Precision* means that the algorithm produces fewer false positives.

#### Recall

*Recall* is defined as the ratio of the number of correctly detected positive samples to the total number of actual positive samples present in the dataset.

3 $$\:Recall=\frac{true\:positive}{true\:positive+false\:negative}\:\:\:\:\:\:\:\:\:\:\:\:\:\:\:\:\:\:\:\:\:\:\:\:$$

where *false negative* refers to the incorrectly identified negative samples. *Recall* measures the algorithm’s ability to discover targets, indicating how many of the actual targets present were correctly detected. A higher *Recall* means that the algorithm produces fewer false negatives.

#### F1-Score

The *F1-Score* is the harmonic mean of *Precision* and *Recall*, integrating the performance of both metrics. Its calculation formula is as follows.

4 $$\:F1=2\times\:\frac{(Precision\times\:Recall)}{Precision+Recall}\:\:$$

the *F1-Score* provides an overall evaluation, helping us to balance the relationship between *Precision* and *Recall*, thereby allowing for a comprehensive assessment of the algorithm’s detection performance.

### Algorithm comparison

Comparative experiments were conducted between the proposed method and several mainstream cylinder detection algorithms. Performance comparisons of each algorithm were made based on the selected simulated and measured datasets, focusing on Precision, Recall, and F1-Score. Three representative comparison algorithms were chosen: CloudCompare^30^, Schnabel’s method^5^, Du‘s method^23^ and Wu‘s method^25^.

CloudCompare is an open-source software widely applied for point cloud processing and analysis, equipped with powerful data handling capabilities and extensive plugin support. Its built-in cylinder detection tool utilizes mature algorithms that can detect cylinders in various complex scenarios. By selecting CloudCompare as a comparison method, the performance and advantages of our method can be directly evaluated against commonly used industry tools, thereby demonstrating the improvements in practicality and usability of our approach. The shape detection algorithm based on RANSAC, proposed by Schnabel et al., is recognized as one of the classic methods for extracting geometric primitives from point clouds. This method efficiently detects basic geometric shapes within a point cloud and is characterized by high computational efficiency and ease of implementation. The algorithm recently proposed by Du et al. has been significantly influential in the field of point cloud cylinder detection, maintaining high detection precision in complex environments. By comparing our method with Du‘s method, improvements in robustness and recall can be assessed, validating its effectiveness in handling real-world data. The comparison methods chosen above all belong to the traditional geometry-driven paradigm. The QuadricsNet method proposed by Wu et al. recently is based on the data-driven deep learning paradigm. The selection of Wu’s method as a comparison can reveal the adaptability differences between traditional geometric constraints and deep learning in the task of cylinder detection. Through the selection of these three comparison methods, a comprehensive evaluation of the proposed algorithm’s performance is aimed for from different perspectives. CloudCompare represents the standard of practical application tools, Du‘s method embodies advanced research results in the academic field, Schnabel’s method serves as a classic method for reference, while Wu’s method serves as a representative of the current popular deep learning approaches. With the exception of the existing software tool CloudCompare and Wu’s method (the authors have provided the source code in Python), all other comparison methods were independently implemented in a MATLAB environment. To ensure the fairness of comparisons, adherence was made as closely as possible to the algorithmic principles and parameter settings presented in the original papers, with the corresponding code being rewritten. By strictly reproducing the algorithms according to the original authors’ ideas and steps, it was ensured that each method was evaluated under the same conditions. This approach helps to eliminate the influence of implementation differences, making the comparison results more objective and credible. Additionally, the generation methodology of the ground truth cylinders is detailed in Appendix C.

**Fig. 7:**
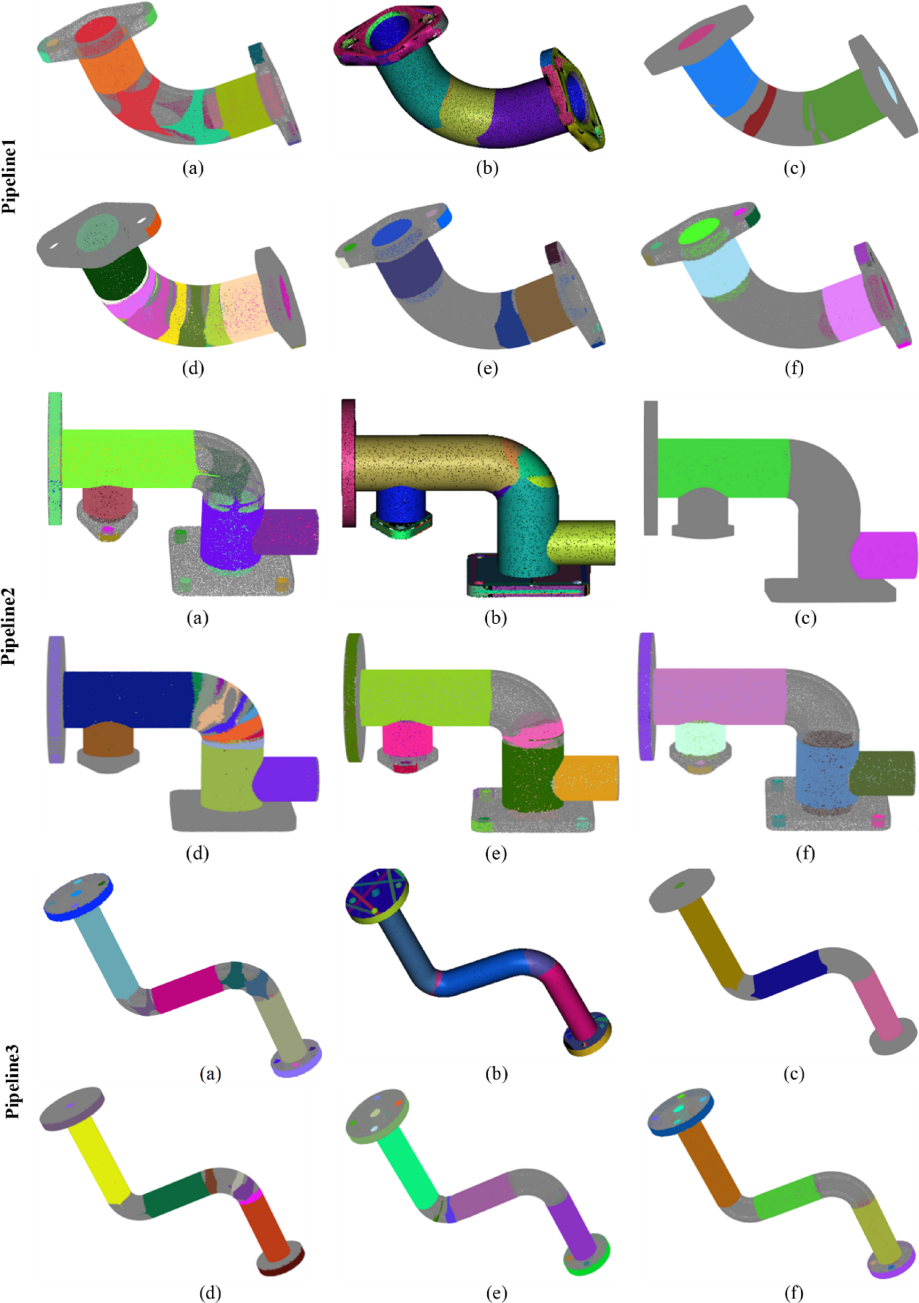

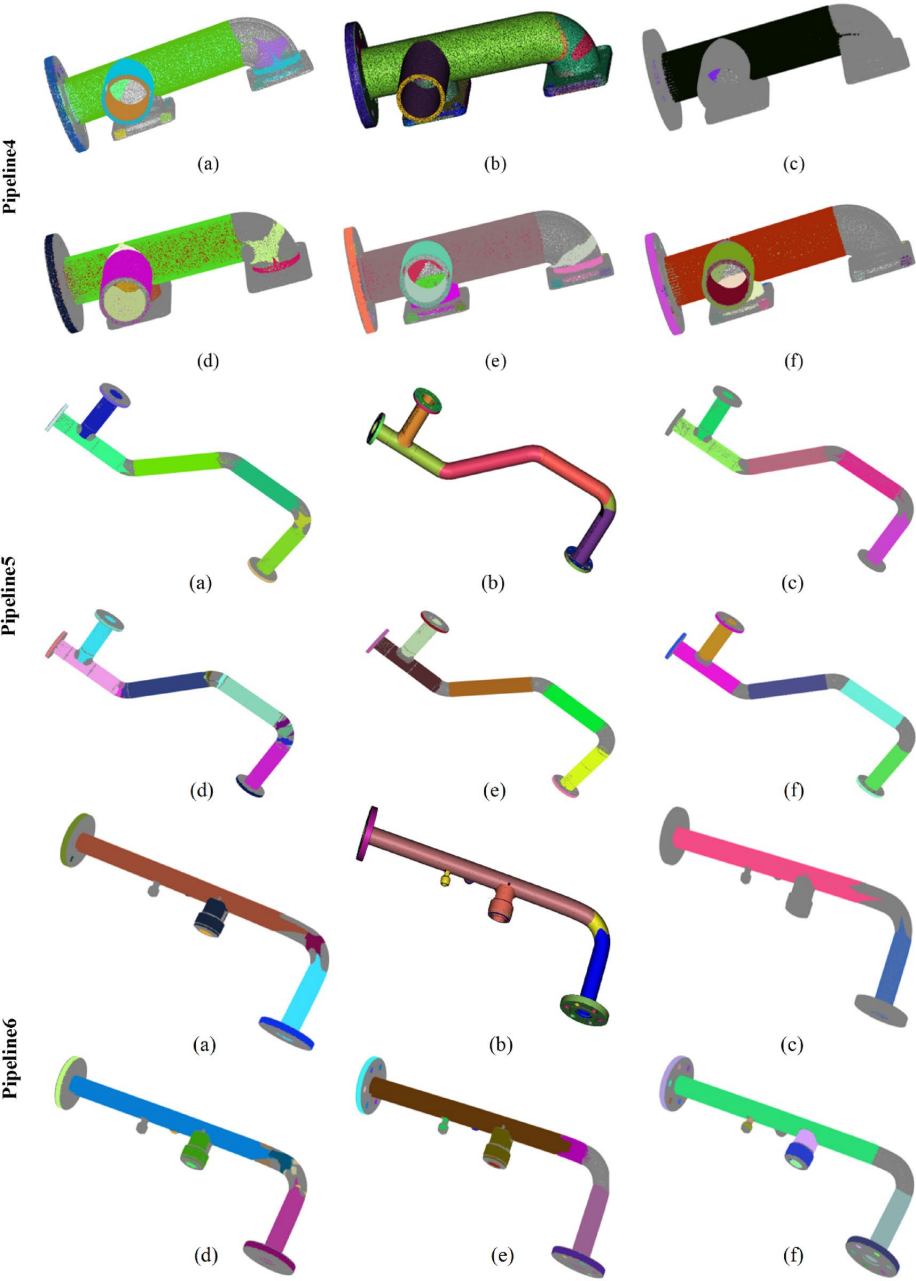

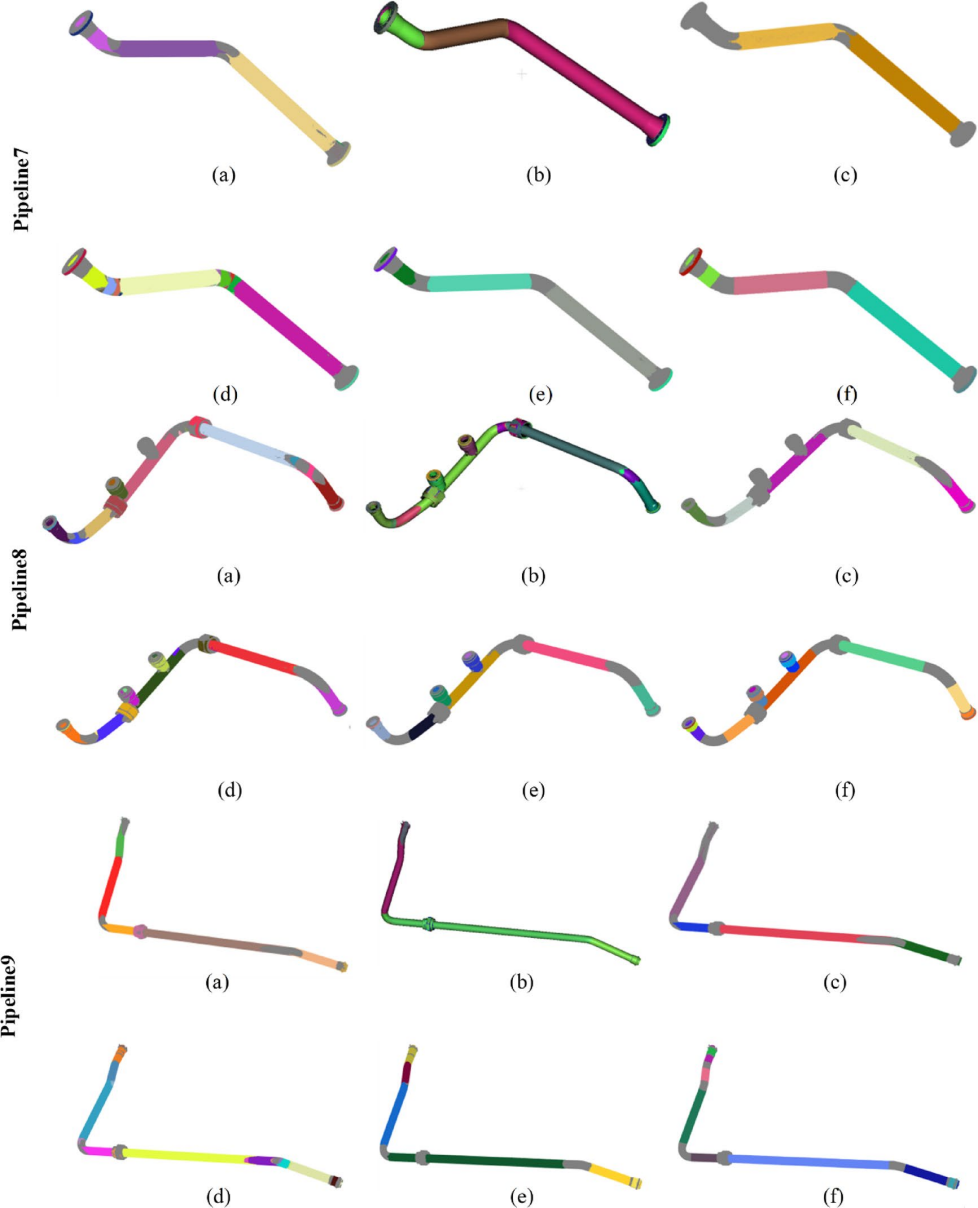
Comparison of detection results on all datasets. (a), (**b**), (**c**), (d), (**e**), and (**f**) represent the detection results of Wu’s method, the CloudCompare software, Du’s method, Schnabel‘s method, our method, and the ground truth, respectively. Different colors highlight different cylinders.

Figure 7 presents a comparison of the performance of different methods across all datasets. Panels (a), (b), (c), (d), (e), and (f) respectively illustrate the detection results of Wu’s method, the CloudCompare software, Du’s method, Schnabel’s method, and our method, along with a benchmark. Different colors emphasize different cylinders. Table 2 provides the detection statistics for each method. The terms ALL, TP, FP, and FN denote the total number of detected cylinders, the number of true positive samples, the number of false positive samples, and the number of false negative samples, respectively. Their specific calculation criteria and methods are presented in Appendix D.

From Fig. 7; Table 2, it can be observed that Wu’s method performs poorly in the detection of bent pipes and flange holes, mainly for the following reasons: The bent sections are usually toroidal surfaces (tori), the curvature variations of which require higher-order parameters for accurate description. However, the quadratic surface model (10 parameters) relied upon by QuadricsNet is incapable of precisely fitting such geometries. As a result, the point clouds of toroidal surfaces are mistakenly segmented into multiple cylinders, leading to false detections. In addition, the flat structures such as flange holes, due to their small proportion in the point cloud and special spatial distribution, cause a decrease in the confidence of the classification module of QuadricsNet. CloudCompare tends to identify as many potential cylindrical features as possible during the detection process, resulting in a high recall rate. This strategy allows most of the actual cylinders to be detected; however, it also introduces a large number of false positives, leading to a lower precision. This indicates that the CloudCompare method exhibits high sensitivity during feature extraction but lacks an effective mechanism for filtering errors and noise. In contrast, Du’s method employs stricter discriminative conditions and threshold settings during the detection process, confirming the presence of cylinders only under highly certain circumstances. This conservative approach effectively reduces the occurrence of false positives, thereby achieving the highest precision. However, overly stringent conditions lead to many actual cylinders not being detected, resulting in almost the lowest recall rate. Schnabel’s method performs poorly across all three performance metrics due to its limited capabilities in feature extraction, parameter estimation, and filtering mechanisms, which result in a high rate of false positives and false negatives. This indicates that this method lacks effective robustness and accuracy when handling real complex point cloud data. In contrast, the proposed method improves robustness in feature extraction, model fitting, and filtering mechanisms, achieving a substantial increase in recall and F1-score while maintaining precision comparable to Du’s method. This suggests that the proposed method not only effectively filters out false positives and maintains high precision but also comprehensively detects actual cylindrical features. The performance advantages can be attributed to several factors. First, an advanced candidate cylinder estimation method was employed, which enhanced the success rate of cylinder detection from the outset. Second, a robust fitting model was introduced during the cylinder fitting process, enabling the adaptive isolation of outlier influences. Lastly, a multi-scale analysis approach was integrated, improving the ability to filter out false arcs that frequently appear in curved sections of pipelines. These improvements collectively enhance the practicality of the algorithm, rendering it superior in performance for complex pipeline applications.

**Table 2:** Performance evaluation on all datasets. ALL, TP, FP, and FN represent, respectively, the total number of detected cylinders, the number of true positives, the number of false positives, and the number of false negatives.

	[ALL TP FP FN]	Precision	Recall	F1
Pipleline1				
Wu Cloudcompare Du Schnabel Ours	[13 10 3 2] [61 10 51 2] [5 4 1 8] [36 4 32 8] [13 12 1 0]	0.7692 0.1639 0.8000 0.1111 0.9231	0.8333 0.8333 0.3333 0.3333 1.0000	0.8000 0.2740 0.4706 0.1667 0.9600
Pipleline2				
Wu Cloudcompare Du Schnabel Ours	[21 16 6 6] [62 17 45 5] [3 3 0 19] [33 7 26 15] [25 21 4 1]	0.7273 0.2742 1.0000 0.2121 0.8400	0.7273 0.7727 0.1364 0.3182 0.9545	0.7273 0.4048 0.2400 0.2545 0.8936
Pipleline3				
Wu Cloudcompare Du Schnabel Ours	[20 10 10 5] [35 13 22 2] [5 5 0 10] [14 7 7 8] [17 15 2 0]	0.5000 0.3714 1.0000 0.5000 0.8824	0.6667 0.8667 0.3333 0.4667 1.0000	0.5714 0.5200 0.5000 0.4828 0.9375
Pipleline4				
Wu Cloudcompare Du Schnabel Ours	[19 18 1 2] [42 16 26 5] [2 2 0 19] [9 5 4 16] [23 21 2 0]	0.9474 0.3810 1.0000 0.5556 0.9130	0.9000 0.7619 0.0952 0.2381 1.0000	0.9231 0.5079 0.1739 0.3333 0.9545
Pipleline5				
Wu Cloudcompare Du Schnabel Ours	[13 12 1 26] [67 32 35 3] [5 5 0 30] [22 8 14 27] [30 30 0 5]	0.9231 0.4776 1.0000 0.3636 1.0000	0.3158 0.9143 0.1429 0.2286 0.8571	0.4706 0.6275 0.2500 0.2807 0.9231
Pipleline6				
Wu Cloudcompare Du Schnabel Ours	[13 8 5 14] [46 11 35 11] [2 2 0 20] [15 5 10 17] [24 17 7 5]	0.6154 0.2391 1.0000 0.3333 0.7083	0.3636 0.5000 0.0909 0.2273 0.7727	0.4571 0.3235 0.1667 0.2703 0.7391
Pipleline7				
Wu Cloudcompare Du Schnabel Ours	[8 6 2 23] [16 7 9 22] [2 2 0 27] [22 5 17 24] [16 16 0 13]	0.7500 0.4375 1.0000 0.2273 1.0000	0.2069 0.2414 0.0690 0.1724 0.5517	0.3243 0.3111 0.1290 0.1961 0.7111
Pipleline8				
Wu Cloudcompare Du Schnabel Ours	[17 6 11 8] [48 2 46 12] [5 3 2 11] [18 5 13 9] [11 7 4 7]	0.3529 0.0417 0.6000 0.2778 0.6364	0.4286 0.1429 0.2143 0.3571 0.5000	0.3871 0.0645 0.3158 0.3125 0.5600
Pipleline9				
Wu Cloudcompare Du Schnabel Ours	[8 5 3 3] [23 1 22 7] [4 4 0 4] [13 6 7 2] [6 5 1 3]	0.6250 0.0435 1.0000 0.4615 0.8333	0.6250 0.1250 0.5000 0.7500 0.6250	0.6250 0.0645 0.6667 0.5714 0.7143
All Piplelines				
Wu Cloudcompare Du Schnabel Ours	[132 91 42 89] [400 109 291 69] [27 25 2 153] [182 52 130 126] [165 144 21 34]	0.6842 0.2725 0.9259 0.2857 0.8727	0.5056 0.6124 0.1404 0.2921 0.8090	0.5815 0.3772 0.2439 0.2889 0.8397

It is essential to quantitatively evaluate the cylinder detection efficiency of each method. The proposed algorithm, Schnabel’s method, and Du’s method are all unsupervised and deterministic algorithms that rely on geometric analysis. In contrast, Wu’s QuadricsNet, as a deep learning-based approach, is fundamentally different because it involves data-driven learning and requires training on a large dataset. Additionally, CloudCompare, as an optimized industrial software, does not provide a fair comparison in terms of computational efficiency due to its specialized design. Therefore, only the proposed method, Schnabel’s method, and Du’s method are compared in terms of computational complexity. The point cloud of Pipeline5, as shown in Fig. 7, is used to evaluate computational complexity. This point cloud contains 2,120,487 points. Each of the three methods was run five times on this point cloud, and the runtimes are presented in Table 3. The average runtime of Schnabel’s method is 3,197 s, and that of Du’s method is 375 s, while the proposed method demonstrates higher efficiency with an average runtime of 283 s. In the proposed method, the introduction of the three-point sampling strategy significantly reduces the number of false candidate cylinders before fitting, thus avoiding subsequent cylinder fitting operations. Moreover, the efficient cylinder fitting method further enhances the overall efficiency.

**Table 3:** Comparison of computational efficiency of cylinder detection Algorithms.

Method	1	2	3	4	5	Avg*(s)
Schnabel’s method	3,325	3,089	3,324	3,525	2,722	3,197
Du’s method	360	344	370	394	407	375
Ours	300	275	288	241	311	283

*Avg is the abbreviation for average runtime.

### Sensitivity analysis of threshold parameters

#### Sensitivity analysis of clustering threshold parameters

Table 4 has been added to demonstrate the impact of the scaling factor n for the clustering threshold T_cluster_ on detection performance. Experiments show that when *n* = 3, the best balance between Recall and F1 score (0.8571/0.9231) is achieved on the Pipeline5 dataset. As n increases from 1 to 4, the F1 value first increases and then decreases, thereby validating the rationality of the formula T_cluster_ =3d_M_.

**Table 4:** The sensitivity experiment of the clustering threshold parameter.

*n*	1	1.5	2	3	3.5	4
Recall	0.7042	0.8152	0.8411	0.8571	0.8428	0.8401
F1	0.7374	0.8623	0.9049	0.9231	0.9128	0.8825

#### Quantitative evaluation of the angle threshold T_β_

In the validation of cylinder detection, the angle threshold T_β_ is employed to constrain the angular relationships between the axes of neighboring cylinders, thereby filtering out false detections that arise in bent pipeline regions. The impact of T_β_ on performance has been tested on Pipeline3 (simulated data with 15 cylinders) and Pipeline7 (real-world data with 29 cylinders), as shown in the Table 5.

**Table 5:** The experiment on the impact of angle threshold T_β_ on FP and FN.

T_β_(^o^)	Pipeline3		Pipeline7	
	FP	FN	FP	FN
1	0	4	1	3
2	2	0	2	0
3	3	0	4	0
5	6	0	7	0

Due to minor assembly errors or point cloud noise in industrial scanning data, strict angular constraints (such as T_β_= 1^o^) lead to a significant increase in false negatives (FN), such as the detection of 4 fewer cylinders in Pipeline3. However, using a too relaxed threshold (such as T_β_= 5^o^) results in a substantial rise in false positives (FP), especially in bent regions, with 7 false cylinders detected in Pipeline7. The optimal value (T_β_= 2^o^) balances false negatives and false positives, achieving the highest F1 score on both simulated and real data. The angle threshold T_β_ effectively distinguishes between true cylinders and false detections in bent pipes by strictly constraining the geometric relationships between neighboring cylinders, while tolerating minor angular deviations caused by scanning noise.

#### The impact of the hyperparameter λ on model performance

An analysis has been conducted on the selection of the hyperparameters λ_1_-λ_4_ for the threshold T_dis_ in Eq. (1) and their impact on model performance. As the radius *r* of the cylinder increases, the discreteness of the intersection coordinates may be amplified due to projection plane errors. Traditional fixed thresholds can lead to false positives for small-radius cylinders or false negatives for large-radius cylinders. By introducing a logarithmic polynomial, T_dis_ can adaptively adjust to changes in radius, balancing the detection sensitivity for cylinders of different sizes. The parameters λ_1_-λ_3_ control the nonlinear response to changes in radius, preventing the threshold from growing exponentially with *r*. λ_4_ serves as a baseline offset to ensure that the threshold remains reasonable even for small radii. The parameters have been optimized on real datasets (Pipeline1-9) with the objective of maximizing the average F1 score. The experimental results are shown in Table 6:

**Table 6:** The impact of the hyperparameter *λ* on model Performance.

Parameter Combination	Precision	Recall	F1
*λ*_i_(The default values)	0.8727	0.8090	0.8397
*λ*_i_(± 10%)	0.8514	0.7938	0.8216
*λ*_i_(± 20%)	0.8121	0.7512	0.7805

As can be seen from Table 6, the F1 score only decreases by about 2% when the hyperparameters λ_i_ are perturbed by ± 10%, indicating that the model is not sensitive to fine-tuning of λ. When the perturbation of the hyperparameters λ_i_ is increased to ± 20%, the F1 score decreases more significantly, by about 7%. In summary, the hyperparameters λ balance detection sensitivity and false detection suppression through experimental optimization and theoretical design, and their adaptive structure is crucial for performance improvement. Experiments on parameter perturbation and validation in multiple scenarios have shown that the model is robust to the selection of λ, making it suitable for complex industrial pipeline detection.

#### Determination of the fitting point threshold T_fit_

**Fig. 8:**
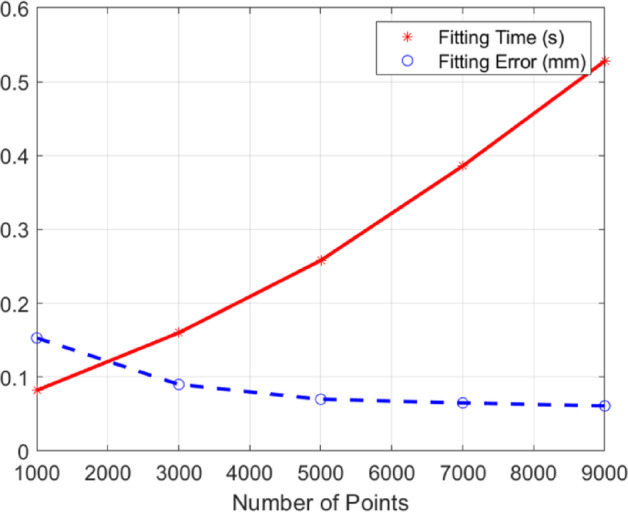
The impact of *T*_*fit*_ on computational cost and fitting error.

The threshold *T*_*fit*_, which defines the maximum number of points used for cylinder fitting, was systematically determined to balance computational efficiency and estimation accuracy. Extensive experiments on both simulated and real-world datasets revealed that setting *T*_*fit*_ = 5000 provides an optimal trade-off. As illustrated in Fig. 8, the fitting time increases approximately linearly with *T*_*fit*_, while the fitting error exhibits a nonlinear relationship, plateauing beyond this point. Consequently, 5000 points were selected as the threshold, as this value consistently maintains the RMS fitting error below 0.1 mm without incurring unnecessary computational overhead, ensuring both the stability of parameter estimation and processing efficiency for typical industrial cylinders.

### Discussion

The proposed method exhibits strong adaptability to variations in pipeline length, diameter, occlusion, and placement configuration. In addressing elongated pipeline structures, the proposed method effectively reduces data complexity through an iterative clustering strategy (Sect. 3.1). Even when long pipelines are initially segmented into multiple sub-regions, the subsequent three-point random sampling for candidate cylinder estimation (Sect. 3.2) and high-precision cylinder fitting (Sect. 3.3) ensure accurate identification and merging of continuous cylindrical structures across sub-regions. From a probabilistic perspective, the likelihood of sampling three points belonging to the same cylinder is significantly higher in elongated pipelines, thereby enhancing the reliability of initial localization. Furthermore, during cylinder fitting, points within a distance of 3σ from the cylindrical surface and with normals aligned to the cylinder axis are validated as inliers (Sect. 3.3). This allows points from different sub-regions (e.g., segmented due to intermediate data loss) to support the fitting of the same cylinder under geometric consistency. Experimental results demonstrate the successful detection of elongated pipeline structures in real-world datasets (Pipelines 6, 7, and 9 in Fig. 7). These pipelines comprise cylindrical segments of varying lengths and spatial configurations, validating the efficacy of the method for elongated pipeline detection.For handling diameter variations, the method adapts autonomously through the radius calculation mechanism in three-point sampling (Sect. 3.2, Algorithm 2). This algorithm computes radii (r₁, r₂, r₃) based on spatial relationships among triple points, enabling the identification of cylindrical structures with differing diameters within the same pipeline. Through multi-detection consistency validation (Sect. 3.4), cylinders maintaining stable radius estimates across iterations are retained, while transitional regions (typically corresponding to conical structures) are naturally excluded due to their non-compliance with the cylindrical equation.In addressing occlusions, the method employs a synergistic mechanism of supporting point cloud extraction and multi-stage filtering. The extraction of locally consistent inliers (Sect. 3.3) ensures reliable cylinder fitting based on locally valid point sets, even under partial occlusion. The multi-detection consistency check (Sect. 3.4) effectively eliminates false detections caused by fragmented inliers resulting from occlusions. Simultaneously, the iterative removal mechanism (Algorithm 1) progressively exposes occluded regions by sequentially removing detected cylinders, thereby facilitating subsequent detection opportunities. Additionally, while our experiments focused on isolated pipelines for validation, the method is applicable to installed pipelines provided sufficient surface visibility is achieved through multi-view scanning. Scenarios with extreme occlusion may require complementary techniques such as topological inference or prior model registration.

**Fig. 9:**
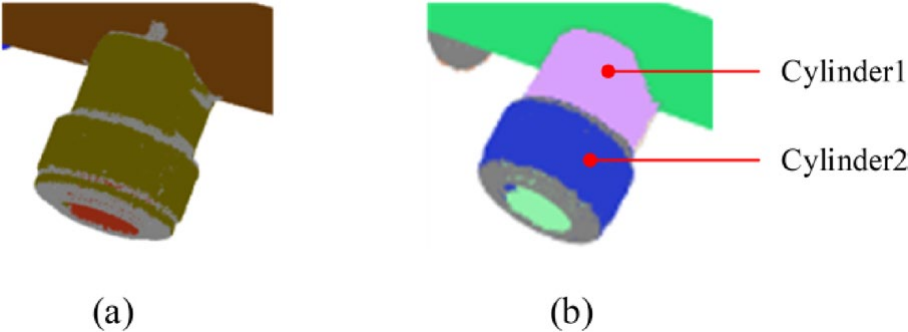
For cylinders with similar diameters and axes, false detections and missed detections occurred. (**a**) The detection results of the proposed method in this paper, (**b**) the ground truth.

Despite the method’s demonstrated adaptability in the aforementioned aspects, some limitations are acknowledged: Firstly, dense occlusions may compromise the completeness of supporting point extraction. Future work will integrate semantic context (e.g., pipeline topology) to infer occluded regions. Secondly, subtle abrupt diameter changes may be misclassified as independent cylinders. This can be mitigated through post-processing incorporating geometric constraints. These directions for improvement will provide more comprehensive solutions for pipeline detection in complex industrial scenarios. Finally, like other methods that rely on normal estimation, the proposed method lacks robustness against high levels of noise. When there is a significant amount of noise present in the point cloud, noise points may interfere with the normal vector estimation, making it challenging for the algorithm to accurately fit the cylindrical model, which can result in false positives or false negatives. Therefore, in practical applications, to ensure the reliability of detection results, it is typically required that the input point cloud data be acquired from industrial-grade precision measurement devices, such as the HyperScan Super, MetraSCAN 3D, and Atos Q scanners. This requirement somewhat limits the applicability of the proposed method. Fortunately, such devices have been widely adopted in the high-end manufacturing industry. Additionally, for cylinders with similar diameters and axes, the proposed method may struggle to differentiate between them, leading to both false positives and false negatives, as illustrated in Fig. 9.

## Conclusion

In this study, a robust method for the automatic detection of cylinders from unstructured scanned point clouds has been proposed, addressing the challenges associated with cylinder detection in complex pipeline scenarios within the high-end manufacturing field. The method utilizes an iterative clustering segmentation strategy to simplify point cloud data processing, thereby enhancing the likelihood of accurately sampling points from the same cylindrical structure. A novel candidate cylinder estimation approach based on three-point random sampling has been introduced, allowing for reliable parameter estimation without imposing restrictive assumptions on cylinder orientation or aspect ratio. The high-precision cylinder fitting algorithm employed in this method significantly improves the localization and validation of detected cylinders, while a multi-filtering mechanism effectively reduces the false positive rate, enhancing the overall robustness and reliability of the detection process. Experimental results indicate that the proposed method achieves excellent performance in terms of precision, recall, and F1 score, demonstrating its effectiveness in both simulated and real-world applications.

Despite its numerous advantages, the method does exhibit limitations, particularly in high-noise environments, where interference with normal vector estimation may lead to inaccuracies in cylinder fitting. To ensure the reliability of detection results, it is recommended that input point cloud data be sourced from high-precision industrial measurement devices. Additionally, the method may struggle to differentiate between cylinders with similar diameters and axes, potentially resulting in false positives and false negatives. Overall, the proposed method showcases significant application potential in the detection of cylinders within complex pipelines, providing a valuable tool for reverse engineering design in the high-end manufacturing industry. Future work will focus on further enhancing the algorithm’s robustness against noise and exploring advanced techniques for distinguishing similar cylindrical structures. Specifically, it will be noted in the revised manuscript that the computational efficiency of the current method under extremely large-scale scenarios remains an area requiring further optimization. Subsequent research efforts will be directed toward algorithm parallelization and optimization, with the validated method subsequently applied to plant-scale piping system point clouds to rigorously evaluate its scalability and practical utility in large-scale operational environments.

## Supplementary Information

Below is the link to the electronic supplementary material.

Supplementary Material 1

## Data Availability

This data is available at the following link: [https://github.com/GCCao/Cylinders\_detection\_Cao\_V2](https:/github.com/GCCao/Cylinders_detection_Cao_V2).

## Code availability

All the code is available at the following link: https://github.com/GCCao/Cylinders_detection_Cao_V2.
