# Supplementary material for: Automated detection of cylindrical structures in complex pipelines using iterative point cloud segmentation and high-precision fitting
Source: Sci Rep. 2025 Nov 27;15:45535. doi: 10.1038/s41598-025-30323-8 (PMC12749517; doi:10.1038/s41598-025-30323-8)
Supplement: Supplementary file 1 — Supplementary Material 1 [file 41598_2025_30323_MOESM1_ESM.docx]

Appendix A: Estimation of *M*

Step1: Calculating the probability that three randomly selected points in a single trial come from the same cylinder

Assuming that the point cloud PC contains N points and includes K cylinders, with an average of n points per cylinder, the probability that three randomly selected points in a single trial come from the same cylinder can be calculated as follows:

1. A cylinder is chosen from the K available cylinders, providing K possible selections.
2. Once a cylinder has been selected, the number of ways to choose three points from the n points within that cylinder is given by combination operator $\left( \begin{matrix} \boldsymbol{n} \\ \boldsymbol{3} \end{matrix} \right)$which is calculated as:

$$\left( \begin{matrix} \boldsymbol{n} \\ \mathbf{3} \end{matrix} \right)\mathbf{=}\frac{\boldsymbol{n}\mathbf{(}\boldsymbol{n}\mathbf{-1)(}\boldsymbol{n}\mathbf{-2)}}{\mathbf{6}}$$

3. The total number of ways to choose three points from all N points in the point cloud is expressed as$\left( \begin{matrix} \boldsymbol{N} \\ \boldsymbol{3} \end{matrix} \right)$:

$$\left( \begin{matrix} \boldsymbol{N} \\ \mathbf{3} \end{matrix} \right)\mathbf{=}\frac{\boldsymbol{N}\mathbf{(}\boldsymbol{N}\mathbf{-1)(}\boldsymbol{N}\mathbf{-2)}}{\mathbf{6}}$$

4. The probability that three randomly selected points in a single attempt come from the same cylinder can be represented as:

$$\boldsymbol{P}\mathbf{=}\frac{\boldsymbol{K}\boldsymbol{\cdot}\left( \begin{matrix} \boldsymbol{n} \\ \mathbf{3} \end{matrix} \right)}{\left( \begin{matrix} \boldsymbol{N} \\ \mathbf{3} \end{matrix} \right)}$$

5. Substituting the combinations into the equation yields:

$$\boldsymbol{P}\mathbf{=}\frac{\boldsymbol{K}\boldsymbol{\cdot}\left( \begin{matrix} \boldsymbol{n} \\ \mathbf{3} \end{matrix} \right)}{\left( \begin{matrix} \boldsymbol{N} \\ \mathbf{3} \end{matrix} \right)}\mathbf{=}\frac{\boldsymbol{K}\boldsymbol{\cdot}\boldsymbol{n}\mathbf{(}\boldsymbol{n}\mathbf{-1)(}\boldsymbol{n}\mathbf{-2)}}{\boldsymbol{N}\mathbf{(}\boldsymbol{N}\mathbf{-1)(}\boldsymbol{N}\mathbf{-2)}}$$

This formula can be used to calculate the probability that three randomly selected points in a single trial come from the same cylinder by substituting the specific values of *N*, *K*, and *n* into the equation.

**Step2: Calculating the maximum number of iterations *M***

In Algorithm 2, after *M* iterations, it is intended that the probability of the selected three points originating from the same cylinder is substantially high, with a requirement of exceeding 99.9999% as specified in this paper. Therefore, the original problem is reformulated to determine the number of iterations necessary to ensure that, given a probability *P* that all three points selected in a single trial originate from the same cylinder, the probability that all three points indeed come from the same cylinder reaches 99.9999%.

1. Setting the objective

The desired probability of success is expressed as:

$$1-\left( 1-P \right)^{M}\geq0.999999$$

This indicates that the probability of at least one successful event must be greater than or equal to 99.9999%.

1. Reformulating the inequality:

This can be restated as:

$$\left( 1-P \right)^{M}\leq0.000001$$

1. Taking the natural logarithm:

by applying the natural logarithm to both sides of the inequality, it can be expressed as:

$$M\ln\left( 1-P \right)\leq ln(0.000001)$$

Since ln(1 - *P*) is a negative value, the direction of the inequality must be reversed:

$$M\geq\frac{ln(0.000001)}{\ln\left( 1-P \right)}$$

By substituting the known value of *P* into this formula, the required number of iterations *M* can be calculated. Due to the fact that the aforementioned derivation does not account for the interference caused by the non-cylindrical sections of the pipeline, it is suggested that the value of *M* in practical applications should be greater than the theoretical value, for instance, approximately double the theoretical value.

**Step3: Example**

Given the *N*, *K*, and *n* are respectively set to 200,000, 40, and 5,000, the probability of selecting three points from the same cylinder *P* is presented as follows.

$$P=\frac{K\cdot n(n-1)(n-2)}{N(N-1)(N-2)}=\frac{40\times5000\times4999\times4998}{200000\times199999\times199998}=0.000625$$

Substituting into the formula regarding *M*:

$$M\geq\frac{\ln\left( 0.000001 \right)}{\ln\left( 1-0.000625 \right)}\approx22111$$

Therefore, it is concluded that a minimum of 22111 iterations is required in this example. It is noted that different values of *P* will result in varying numbers of required iterations. According to the previous usage recommendations, the value of *M* may be approximated as twice the theoretical value. Consequently, a value of *M* of 45,000 is deemed appropriate in this example.

Appendix B: Derivation of the Analytical Expression for the Cylinder

The distance from each point on the surface of the cylinder to the axis is regarded as equal to the radius. Consequently, the analytical expression of the cylinder is derived based on this property, as follows:

**Step 1: Set the Axis Line Equation**

The axis of the cylinder is represented by a line equation. An explicit parametric equation is not required; instead, points and vectors are introduced to represent this line. The axis is defined using any point on the line and its direction vector. The distance from any point **p**=(*x*, *y*, *z*) to the line **c**+t**V** is to be calculated.

**Step 2: Construct the Distance Formula**

All points **p**=(*x*, *y*, *z*) for which the distance to the axis equals *r* are sought. This can be expressed using vectors and dot products.

**Step 3: Find the Expression for Distance**

Let **c**=(*a*,*b*,*c*) represent a point on the axis of the cylinder, and let **V**=(*d*, *e*, *f*) denote the direction vector of the axis. The vector **p**=(*x*, *y*, *z*) is defined, and thus the vector from **c** to **p** is expressed as **p**−**c**=(*x−a*, *y−b*, *z−c*).

**Step 4: Calculate the Distance**

The distance from the line segment to the line is expressed using the projection of the vector. First, the projection of **p**−**c** onto **V** is to be computed:

$${Projection}_{\mathbf{V}}(\mathbf{p}-\mathbf{c})=\frac{(\mathbf{p}-\mathbf{c})\cdot\mathbf{V}}{{||\mathbf{V}||}^{2}}\mathbf{V}$$

Here,

$$\left| \left| \mathbf{V} \right| \right|=\sqrt{d^{2}+e^{2}+f^{2}}$$

**Step 5. Determine the Distance**

Thus, the distance D can be expressed as:

$$D=||\left( \mathbf{p}-\mathbf{c} \right)-{Projection}_{\mathbf{V}}(\mathbf{p}-\mathbf{c})||$$

This distance should equal *r*.

**Step 6. Algebraic Form**

The equation is obtained by rearranging the above expression:

$${||\mathbf{p}-\mathbf{c}||}^{2}-\left( \frac{\left( \mathbf{p}-\mathbf{c} \right)\cdot\mathbf{V}}{\left| \left| \mathbf{V} \right| \right|} \right)^{2}=r^{2}$$

It is expressed in a more specific form:

$$\left( \sqrt{{(x-a)}^{2}+{(y-b)}^{2}+{(z-c)}^{2}} \right)^{2}-\left( \frac{\left( x-a \right)d+\left( y-b \right)e+(z-c)f}{\sqrt{d^{2}+e^{2}+f^{2}}} \right)^{2}=r^{2}$$

**Step 7. Final Expression for the Cylinder**

Finally, the equation for the cylinder is obtained as follows:

$$\sqrt{{(x-a)}^{2}+{(y-b)}^{2}+{(z-c)}^{2}-\frac{\left( \left( x-a \right)d+\left( y-b \right)e+\left( z-c \right)f \right)^{2}}{d^{2}+e^{2}+f^{2}}}-r=0$$

This equation is described as representing the set of points in space that are all at a distance equal to r from the axis (defined by point **c** and direction vector **V**), thereby defining a cylinder that extends along the axis.

Appendix C: Methodology for Generating Cylindrical Ground Truth

This appendix details the workflow for generating ground truth cylindrical segmentations using PolyWorks software (version 2020). The process combines CAD-guided alignment, semi-automated feature extraction, and manual validation to ensure high accuracy and consistency.

**Step 1: Data Preparation and Alignment**

Import: The original scanned point cloud and the corresponding CAD model (STEP format) of the pipeline were imported into PolyWorks.

Alignment: A best-fit alignment between the point cloud and the CAD model was performed using PolyWorks’Data-to-Reference tool. This ensures spatial consistency between the scanned data and the CAD model, leveraging the latter as a geometric reference.

**Step 2: Cylindrical Feature Extraction**

Manual Segmentation: Cylindrical regions in the point cloud were manually identified and labeled by aligning with the CAD model’s cylindrical components (e.g., pipe bodies, flanges).

Measurement Extraction: For each labeled cylindrical region, PolyWorks’ Extract Measurements tool was used to isolate the points belonging to the cylinder. These points were exported as separate TXT files.

**Step 3: Validation and Refinement**

Visual Inspection: Each extracted cylinder was visually validated against the CAD model to ensure accuracy.

Outlier Removal: Non-cylindrical points (e.g., noise, adjacent structures) were manually removed.

**Step 4: Data Labeling and Integration**

Color Coding: Each cylinder was assigned a unique RGB color (e.g., red, blue) for visualization.

Final Integration: All labeled point clouds were combined into a single TXT file, forming the ground truth dataset.

Appendix D: Specific Calculation Criteria and Methods for ALL, TP, FP, and FN

In order to clarify the statistical process of ALL (total detected cylinders), TP (true positives), FP (false positives), and FN (false negatives) in the experiments, the calculation criteria and methods for each metric are detailed in this appendix.

**Step 1: Definition of Metrics and Calculation Criteria**

1. Basic Definitions

ALL (Total Detection Count): The number of all candidate cylinders output by the algorithm.

TP (True Positives): The number of correctly detected real cylinders (geometrically matched with the labeled cylinders).

FP (False Positives): The number of incorrectly detected cylinders (no real correspondence or parameters exceeding tolerance).

FN (False Negatives): The number of real cylinders that were not detected.

1. Geometric Matching Criteria

To determine that the detected cylinder C_det_ matches the labeled cylinder C_GT_ (i.e., TP), the following conditions must be met:

Axial Deviation: The angle $\emptyset$ between the axes of the two cylinders must be $\emptyset\leq2^{^{\circ}}$

Relative Radius Error: ∣r_det_−r_GT_∣/r_GT_≤5%。

Center Distance: The Euclidean distance d_E_ between the centers of the two cylinders must be ≤5%⋅r_GT_ (to avoid overly strict absolute distance for large-radius cylinders).

1. Matching Process

One-to-One Matching: The Hungarian algorithm is used to match the detection results with the labels one by one, based on minimizing the comprehensive cost:

$$\mathrm{Cost}=w_{1}\cdot\emptyset+w_{2}\cdot\frac{\left| r_{det}-r_{GT} \right|}{r_{GT}}+w_{3}\cdot\frac{d_{E}}{r_{GT}}$$

Here, w_1_, w_2_, and w_3_ are weighting coefficients, which can be adjusted according to the requirements of the task. In this paper, they are set to 0.3, 0.3, and 0.4, respectively.

Basis for Tolerance Thresholds: The thresholds are determined based on industrial pipeline reverse engineering standards (such as ASME B16.9) and verified through synthetic data experiments to ensure a balance between sensitivity and specificity.

**Step 2: Detailed Statistical Process**

1. Calculation of ALL

The total number of candidate cylinders output by the algorithm is directly counted.

1. Calculation of TP

Matching pairs are filtered through the above geometric criteria using the Hungarian algorithm, ensuring that each C_det_ matches only one C_GT_ to avoid double counting.

Example: If a labeled cylinder is detected three times, only the optimal match is counted as TP, and the others are counted as FP.

1. Calculation of FP

Detection results that do not meet the geometric matching criteria.

Redundant detections remaining after matching (e.g., repeated detection of the same real cylinder).

1. Calculation of FN

The number of labeled cylinders that are not matched by any detection result.
